# First Steps toward Harmonized Human Biomonitoring in Europe: Demonstration Project to Perform Human Biomonitoring on a European Scale

**DOI:** 10.1289/ehp.1408616

**Published:** 2014-12-11

**Authors:** Elly Den Hond, Eva Govarts, Hanny Willems, Roel Smolders, Ludwine Casteleyn, Marike Kolossa-Gehring, Gerda Schwedler, Margarete Seiwert, Ulrike Fiddicke, Argelia Castaño, Marta Esteban, Jürgen Angerer, Holger M. Koch, Birgit K. Schindler, Ovnair Sepai, Karen Exley, Louis Bloemen, Milena Horvat, Lisbeth E. Knudsen, Anke Joas, Reinhard Joas, Pierre Biot, Dominique Aerts, Gudrun Koppen, Andromachi Katsonouri, Adamos Hadjipanayis, Andrea Krskova, Marek Maly, Thit A. Mørck, Peter Rudnai, Szilvia Kozepesy, Maurice Mulcahy, Rory Mannion, Arno C. Gutleb, Marc E. Fischer, Danuta Ligocka, Marek Jakubowski, M. Fátima Reis, Sónia Namorado, Anca Elena Gurzau, Ioana-Rodica Lupsa, Katarina Halzlova, Michal Jajcaj, Darja Mazej, Janja Snoj Tratnik, Ana López, Estrella Lopez, Marika Berglund, Kristin Larsson, Andrea Lehmann, Pierre Crettaz, Greet Schoeters

**Affiliations:** 1 Unit Environmental Risk and Health, Flemish Institute for Technological Research (VITO), Mol, Belgium; 2 University of Leuven, Leuven, Belgium; 3 Umweltbundesamt (UBA), Berlin, Germany; 4 Instituto de Salud Carlos III, Majadahonda (Madrid), Spain; 5 Institute for Prevention and Occupational Medicine of the German Social Accident Insurance, Institute of the Ruhr-Universität Bochum (IPA), Bochum, Germany; 6 Public Health England, Chilton, United Kingdom; 7 Environmental Health Sciences International, Hulst, the Netherlands; 8 Jožef Stefan Institute, Ljubljana, Slovenia; 9 University of Copenhagen, Copenhagen, Denmark; 10 BiPRO GmbH, Munich, Germany; 11 Federal Public Service (FPS) Health, Food Chain Safety and Environment, Brussels, Belgium; 12 State General Laboratory, Nicosia, Cyprus; 13 Paediatric Clinic, Larnaca General Hospital, Larnaca, Cyprus; 14 National Institute of Public Health, Praha, Czech Republic; 15 National Institute of Environmental Health, Budapest, Hungary; 16 Health Service Executive, Dublin, Ireland; 17 Luxembourg Institute of Science and Technology (LIST), Belvaux, Luxembourg; 18 Laboratoire National de Santé, Dudelange, Luxembourg; 19 Nofer Institute of Occupational Medicine, Lodz, Poland; 20 Faculdade de Medicina da Universidade de Lisboa, Lisbon, Portugal; 21 Environmental Health Center, Cluj-Napoca, Romania; 22 Urad Verejneho Zdravotnictva Slovenskej Republiky, Bratislava, Slovakia; 23 Karolinska Institutet, Stockholm, Sweden; 24 Federal Office of Public Health (FOPH), Bern, Switzerland; 25 University of Antwerp, Antwerpen, Belgium; 26 University of Southern Denmark, Odense, Denmark

## Abstract

**Background:**

For Europe as a whole, data on internal exposure to environmental chemicals do not yet exist. Characterization of the internal individual chemical environment is expected to enhance understanding of the environmental threats to health.

**Objectives:**

We developed and applied a harmonized protocol to collect comparable human biomonitoring data all over Europe.

**Methods:**

In 17 European countries, we measured mercury in hair and cotinine, phthalate metabolites, and cadmium in urine of 1,844 children (5–11 years of age) and their mothers. Specimens were collected over a 5-month period in 2011–2012. We obtained information on personal characteristics, environment, and lifestyle. We used the resulting database to compare concentrations of exposure biomarkers within Europe, to identify determinants of exposure, and to compare exposure biomarkers with health-based guidelines.

**Results:**

Biomarker concentrations showed a wide variability in the European population. However, levels in children and mothers were highly correlated. Most biomarker concentrations were below the health-based guidance values.

**Conclusions:**

We have taken the first steps to assess personal chemical exposures in Europe as a whole. Key success factors were the harmonized protocol development, intensive training and capacity building for field work, chemical analysis and communication, as well as stringent quality control programs for chemical and data analysis. Our project demonstrates the feasibility of a Europe-wide human biomonitoring framework to support the decision-making process of environmental measures to protect public health.

**Citation:**

Den Hond E, Govarts E, Willems H, Smolders R, Casteleyn L, Kolossa-Gehring M, Schwedler G, Seiwert M, Fiddicke U, Castaño A, Esteban M, Angerer J, Koch HM, Schindler BK, Sepai O, Exley K, Bloemen L, Horvat M, Knudsen LE, Joas A, Joas R, Biot P, Aerts D, Koppen G, Katsonouri A, Hadjipanayis A, Krskova A, Maly M, Mørck TA, Rudnai P, Kozepesy S, Mulcahy M, Mannion R, Gutleb AC, Fischer ME, Ligocka D, Jakubowski M, Reis MF, Namorado S, Gurzau AE, Lupsa IR, Halzlova K, Jajcaj M, Mazej D, Snoj Tratnik J, López A, Lopez E, Berglund M, Larsson K, Lehmann A, Crettaz P, Schoeters G. 2015. First steps toward harmonized human biomonitoring in Europe: demonstration project to perform human biomonitoring on a European scale. Environ Health Perspect 123:255–263; http://dx.doi.org/10.1289/ehp.1408616

## Introduction

Human biomonitoring (HBM) measures the levels of environmental chemicals or their metabolites in easily accessible body fluids and tissues (Angerer et al. 2006), and reflects all routes of uptake—oral, dermal, inhalative—and all relevant sources. The power of HBM to identify spatial and temporal trends in human exposures has contributed successfully to initiate policy measures and to focus on protection of susceptible populations such as children and pregnant mothers. The ban of lead from gasoline was triggered by elevated blood lead levels in the National Health and Nutrition Examination Survey (NHANES) (Pirkle et al. 1994). Results of the German Environmental Survey (GerES) led to recommendations to avoid mercury-containing amalgam teeth fillings in children (Becker et al. 2013) and contributed to the restriction of phthalate use in plastics (Göen et al. 2011). Increasing levels of polybrominated diphenyl ethers (PBDEs) in maternal milk samples of Sweden have led to the gradual phasing out of lower brominated congeners of PBDEs (Meironyté et al. 1999).

Experience with human biomonitoring in the general population differs among European countries, with long-standing traditions in countries such as Germany (Becker et al. 2008), France (Fréry et al. 2012), the Czech Republic (Cerná et al. 2012), Belgium (Flanders) (Schoeters et al. 2012), and Spain (Pérez-Gómez 2013), whereas other countries have no experience at all.

The “European Environment and Health Action Plan” (European Commission 2004) prioritized the need to harmonize HBM in Europe to allow comparison of data among countries and provide tools for follow-up of temporal and spatial trends in chemical exposures. The preparation of the protocol, including the selection of chemicals and study populations, started in 2005 with the Expert team to Support BIOmonitoring in Europe (ESBIO) project. With the funding of the Consortium to Perform Human Biomonitoring on a European Scale (COPHES) and its demonstration project DEMOCOPHES, the feasibility of a harmonized HBM approach was tested (Joas et al. 2012). COPHES designed the final protocol and made justified choices for exposure biomarkers, sample size, and recruitment strategy. DEMOCOPHES allowed 17 European countries to put this protocol into practice. Selected chemicals included phthalates that are present in some consumer products and food packaging (Koch and Calafat 2009), mercury and cadmium as ubiquitous developmental toxicants of concern (Grandjean and Landrigan 2006), and urinary cotinine (Avila-Tang et al. 2013) as a biomarker for exposure to cigarette smoke; urinary creatinine was included as a measure for urine dilution. Young children and mothers of childbearing age were selected as vulnerable populations. Mercury in hair (Budtz-Jørgensen et al. 2004) and urinary cadmium (Akerstrom et al. 2013) are markers of chemicals that accumulate in the body over a longer time period; urinary phthalate metabolites (Wittassek et al. 2011) and cotinine (Avila-Tang et al. 2013) measured in spot urine samples represent short-term exposure.

## Methods

*Study design and participants*. The cross-sectional survey was designed to include 120 children (5–11 years of age) and their mothers in each country, with 60 mother–child pairs each in Cyprus and Luxembourg because of the countries’ smaller populations. We sampled the children and mothers between September 2011 and February 2012, either through schools or population registries. These were convenience samples with equal shares in an urban and a rural location as defined according to regional standards. We included only healthy children and mothers (no metabolic disturbances), who had sufficient knowledge of the local language and had been living at least for 5 years at the sampling location. Details and rationale for the study design are reported by Becker et al. (2014). The sample size allowed us to estimate preliminary country-specific reference values (Poulsen et al. 1997) and a minimally important difference in mean biomarker values of 30% between countries (α = 0.05, β = 0.80). Fieldworkers from the national study centers were trained and instructions were provided centrally and adapted at national level to each country’s language, cultural conventions, and ethical and legal requirements. Information on characteristics of the study population and potential determinants of internal exposure was obtained through personalized interviews using questionnaires. Standard operation procedures (SOPs) to collect hair and morning urine samples were implemented (Becker et al. 2014). The study was approved by ethics committees in each country (for a list of ethics committees per country, see Supplemental Material, Table S1); mothers and children gave written informed consent or assent, respectively. All procedures followed the national data protection requirements including notification to data protection authorities.

*Chemical analysis*. We established a Quality Assurance Program to guarantee the quality and comparability of analytical results among laboratories (Schindler et al. 2014). Each participating laboratory received SOPs for sampling, sample conservation, and chemical analysis (Becker et al. 2014; Schindler et al. 2014). We organized two interlaboratory comparison investigations and two external quality assessment schemes (ICI/EQUAS) with native control material (hair, urine) sent to all laboratories willing to participate. To evaluate the ICIs, we calculated consensus values as the mean of the results of the participating laboratories (after exclusion of outliers). To evaluate the EQUAS, we calculated assigned values (target values) from the results of experienced, renowned reference laboratories. Laboratories were defined as “qualified laboratories” if they participated successfully in at least one ICI and one EQUAS round or in two EQUAS rounds (Schindler et al. 2014). The number of laboratories that qualified for each analyte was as follows: mercury, 15; cotinine, 9; cadmium, 14; phthalate metabolites [monoethylhexyl phthalate (MEHP), 2-ethyl-5-hydroxyhexyl phthalate (5OH-MEPH), 2-ethyl-5-oxohexyl phthalate (5oxo-MEHP), monoethyl phthalate (MEP), monobenzyl phthalate (MBzP), mono-*n*-butyl phthalate (MnBP), monoisobutyl phthalate (MiBP)], 7; and creatinine, 14.

*Database management and statistical analysis*. National data centers applied uniform rules for database construction by using one centrally developed code book with predefined variable names, unities, formats, and coding rules. Quality controls on the data were performed with centrally developed programs (SAS or SPSS). These strict and uniform rules for database construction allowed us to pool all country-specific data into one central European database. We used SAS software, version 9.3 (SAS Institute Inc.) for analysis of the central database. We replaced values below the LOQ by LOQ/2 and transformed biomarker data to natural log-transformed concentrations (ln). We excluded samples with creatinine concentrations < 300 mg/L or > 3,000 mg/L from statistical analysis [World Health Organization (WHO) 1996]. We calculated weighted geometric means (GM) [95% confidence intervals (CIs)] and 90th percentiles (P90) (95% CI) so that the countries were equally represented, except for Cyprus and Luxembourg, which contributed only half. Using multiple mixed regression models with country as random factor, we identified determinants of exposure biomarkers by including pre-specified confounders and significant covariates (*p* < 0.25 from univariate model to enter and *p* < 0.05 to stay) in a stepwise model. We expressed urinary biomarkers in micrograms per liter with urinary creatinine included as confounder. We expressed results as percent change (95% CI) of biomarker concentration for change of the determinant, after adjustment for all other variables in the model. (For detailed methodology and full models, see Supplemental Material, “Identification of determinants of exposure,” “Comparison of results between countries,” and Table S2.)

**Figure 1:**
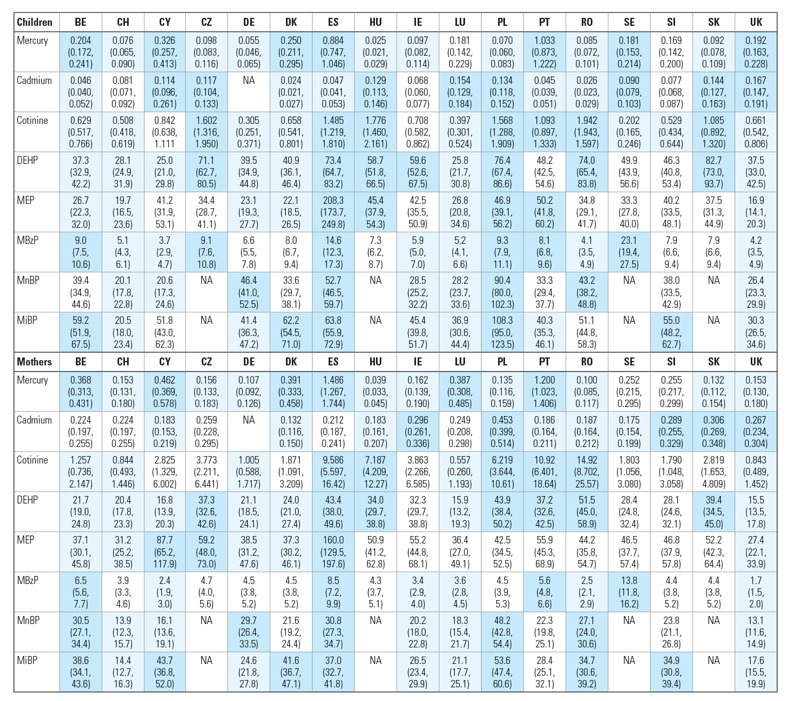
Overview of GMs (95% CIs) of biomarker concentrations (μg/L for urinary markers and μg/g for mercury in hair) in children and mothers of the participating countries. Country codes: BE, Belgium; CH, Switzerland; CY, Cyprus; CZ, Czech Republic; DE, Germany; DK, Denmark; ES, Spain; HU, Hungary; IE, Ireland; LU, Luxembourg; PL, Poland; PT, Portugal; RO, Romania; SE, Sweden; SI, Slovenia; SK, Slovak Republic; UK, United Kingdom. NA, no biomarker data available. For phthalate abbeviations, see Table 2. All data for children are adjusted for age and sex; urinary metabolites are additionally adjusted for urinary creatinine; all data in mothers are adjusted for age; urinary metabolites are additionally adjusted for urinary creatinine; urinary cadmium is additionally adjusted for smoking. Light blue: GM of country significantly below European GM. Dark blue: GM of country is significantly above European GM. White: no significant difference between GM of country and European GM. For European GMs, see Table 2.

**Figure 2:**
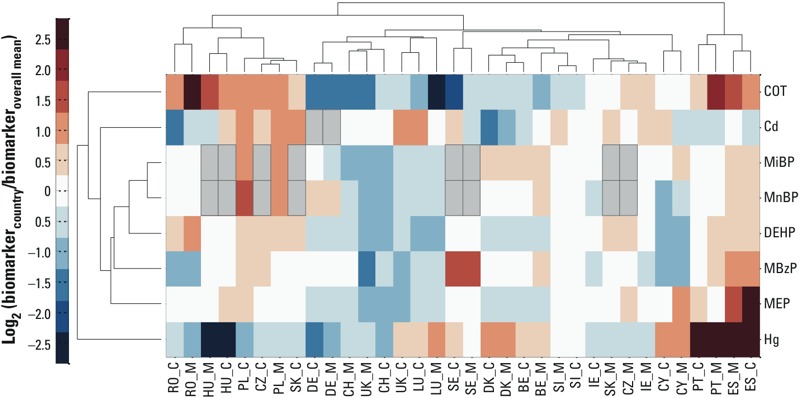
Heat map showing clustering of biomarkers (dendrogram to the left side) and clustering of countries (dendrogram at the top). Red and blue intensities indicate fold increases and decreases, respectively (expressed as log_2_) in country-specific biomarker concentrations adjusted for age and sex relative to the European geometric mean. Abbreviations: Cd, cadmium; COT, cotinine; Hg, mercury. For country codes, see Figure 1. Country codes followed by M present concentrations in mothers; country codes followed by C present concentrations in children. Gray rectangles bordered in black lines indicate missing data.

To compare biomarker values among countries, we compared the GM of a country with the European GM by mixed linear regression analysis, after adjustment for prespecified confounders (Figure 1). To visualize similarity between the biomarker levels and between different countries and/or mothers and children from the same country, we generated a heat map using the clustergram function (Matlab, MathWorks Inc.) (Figure 2). Hierarchical clustering with Euclidean distance metric and average linkage was used to generate the hierarchical tree. Before analysis, the GM of each country was divided by the European GM. The ratio was calculated for mothers and children separately and was logarithmically transformed (log_2_ base) to obtain symmetry around 0 [= log_2_(1)]. The nearest-neighbor method was applied to impute missing data.

To put the results in a health risk context, we calculated the proportion of individuals with levels above health-based guidance values (Aylward et al. 2009a, 2009b; Hays et al. 2008; Schulz et al. 2012; WHO 2004).

## Results

*Determinants of biomarker concentrations*. Descriptive statistics of 1,844 children and mothers included in the study are given in Table 1. Participants were equally recruited according to predefined strata of sex, age, and sampling area in each country. For descriptive statistics of the biomarkers and multiple regression models, see Supplemental Material, Tables S3–S19.

**Table 1:** Descriptive statistics of the study population.

	Children			Mothers		
	*n*	Median (P25–P75)	Minimum–maximum	*n*	Median (P25–P75)	Minimum–maximum
Age (years)	1,844	8 (7, 10)	5–12	1,844	39 (35, 42)	24–52
Urinary creatinine (mg/L)	1,842	1,053 (784, 1,426)	10–3,120	1,839	1,163 (781, 1,618)	57–3,670
Body height (cm)	1,819	135 (127, 145)	98–170	1,836	166 (161, 170)	145–191
Body weight (kg)	1,820	30 (25, 36)	14–81	1,836	64 (58, 72)	35–186
Body-mass index (kg/m²)	1,811	16.3 (14.9, 18.2)	10.0–36.1	1,833	23.2 (21.1, 26.3)	14.7–62.2

	Children			Mothers		
	*n*	Category	*n* (%)	*n*	Category	*n* (%)
Sex	1,844	Boy	912 (49.5)	1,844	Woman	1,844 (100)
		Girl	932 (50.5)			
Area of residence	1,844	Rural	923 (50.1)	1,844	*As in children*	
		Urban	921 (49.9)			
Highest educational level of the family	1,843	Primary (ISCED 0–2)	166 (9.0)	1,843	*As in children*
		Secondary (ISCED 3–4)	607 (32.9)		
		Tertiary (ISCED 5–6)	1,070 (58.1)		
Smoking habits	1,844	Smoker	0 (0)	1,844	Daily smoker	283 (15.3)
		Nonsmoker	1,844 (100)		Occasional smoker	106 (5.7)
					Former smoker	401 (21.7)
					Never smoker	1,054 (57.2)
ETS at home (nonsmokers only)	1,842	Daily	179 (9.7)	1,450	Yes	162 (11.2)
		Less than daily	130 (7.1)		No	1,288 (88.8)
		Never	1,533 (83.2)			
ETS elsewhere (nonsmokers only)	1,842	Yes	775 (42.1)	1,455	Yes	827 (56.8)
		No	1,067 (57.9)		No	628 (43.2)
ETS in last 24 hr (nonsmokers only)	1,840	Yes	232 (12.6)	1,450	Yes	164 (11.3)
		No	1,608 (87.4)		No	1,286 (88.7)

	Children			Mothers		
	*n*	Category	*n* (%)	*n*	Category	*n* (%)
Fish consumption (all types)	1,844	Several times/week	442 (24.0)	1,844	Several times/week	483 (26.2)
		Once a week or less	1,402 (76.0)		Once a week or less	1,361 (73.8)
Consumption of sea fish	1,840	Several times/week	283 (15.4)	1,840	Several times/week	294 (16.0)
		Once a week or less	1,557 (84.6)		Once a week or less	1,546 (84.0)
Consumption of shellfish	1,820	Several times/week	194 (10.7)	1,826	Several times/week	355 (19.4)
		Once a week or less	1,626 (89.3)		Once a week or less	1,471 (80.6)
Consumption of freshwater fish	1,815	Several times/week	248 (13.7)	1,818	Several times/week	298 (16.4)
		Once a week or less	1,567 (86.3)		Once a week or less	1,520 (83.6)
Consumption of seafood products	1,811	Several times/month	94 (5.2)	1,811	Several times/month	154 (8.5)
		Once a month or less	1,717 (94.8)		Once a month or less	1,657 (91.5)
Consumption of ice cream	1,821	Several times/week	185 (10.2)	1,829	Several times/month	536 (29.3)
		Once a week or less	1,636 (89.8)		Once a month or less	1,293 (70.7)
Consumption of chewing gum	1,662	Several times/week	578 (34.8)	1,675	Several times/week	626 (37.4)
		Once a week or less	1,084 (65.2)		Once a week or less	1,049 (62.6)
Use of personal care products^*a*^	1,816	High or moderate	822 (45.3)	1,806	High	861 (47.7)
		Low	994 (54.7)		Moderate or low	945 (52.3)
PVC in house	1,773	PVC in floors or walls	342 (19.3)	1,773	*As in children*	
		No PVC	1,431 (80.7)			
Abbreviations: ETS, environmental tobacco smoke; ISCED, International Standard Classification of Education; P25, 25th percentile; P75, 75th percentile; PVC, polyvinyl chloride. ^***a***^Use of personal care products (PCPs) is calculated as a score based on the frequency (never to daily) of nine PCP groups (makeup, eye makeup, shampoo, hair-styling products, body lotions and creams, fragrances, deodorant, massage oil, and nail polish).						

Fish consumption was the major predictor of mercury levels in hair, both in children and in mothers (see Supplemental Material, Tables S4 and S5). Consumption of sea fish, shellfish, or freshwater fish in the preceding 4 weeks independently contributed to mercury levels in the body. In multiple regression models, frequent (several times/week) compared to sporadic (once/week or less) sea fish consumption was associated with 46% (95% CI: 26, 69%) higher mercury levels in children and 51% (95% CI: 34, 71%) in mothers; shellfish with 56% (95% CI: 35, 79%) in children and 38% (95% CI: 24, 55%) in mothers, freshwater fish with 23% (95% CI: 8, 39%) in children and 23% (95% CI: 11, 37%) in mothers. The GM mercury levels of mothers were higher than those of the children (Table 2), but levels of mothers and children were highly correlated (Spearman’s *r* = 0.72, *p* < 0.001, *n* = 1,833). Older mothers had 15% (95% CI: 5, 24%) higher levels compared to the youngest age group (see Supplemental Material, Table S5). Younger children of 5–8 years showed 8% (95% CI: 0, 17%) higher levels compared with the older group of 9–11 years (see Supplemental Material, Table S4). Participants from families with a higher educational level (tertiary vs. primary education) had 19% (95% CI: 4, 31%) higher levels of mercury in children and 25% (95% CI: 13, 36%) in mothers.

**Table 2:** European exposure values in children and mothers in the COPHES/DEMOCOPHES study and for those in NHANES.

Biomarker of exposure	COPHES/DEMOCOPHES study					NHANES^*d*^			
	*n*	% > LOQ^*a*^	GM (95% CI)^*b*^	P90 (95% CI)^*b*^	*n *(%) exceeding guidance value^*c*^	Period	*n*	GM (95% CI)	P90 (95% CI)
Children
Mercury in hair (μg/g)	1,836	85.9	0.145 (0.139, 0.151)	0.800 (0.698, 0.917)	JECFA: *n *= 25 (1.4)	1999–2000	838	0.12 (0.10, 0.12)	0.41
Urinary cotinine (μg/L)	1,818	57.6	0.80 (0.76, 0.84)	4.90 (3.90, 6.16)	—	—	—	—	—
Urinary cadmium (μg/L)	1,698	70.1	0.071 (0.069, 0.074)	0.220 (0.209, 0.232)	HBM-I: *n *= 6 (0.4) HBM–II: *n *= 0 (0.0) BE: *n *= 0 (0.0)	2009–2010	415	0.057 (0.053, 0.061)	0.130 (0.120, 0.160)
Urinary DEHP metabolites (μg/L)^*e*^	1,816	85.6	47.6 (46.0, 49.3)	137 (126, 150)	HBM-I: *n *= 12 (0.6) BE: *n *= 53 (2.9)	2009–2010	415	MEHP: 1.64 (1.45, 1.85) 5OH-MEHP: 15.0 (13.2, 17.1) 5-oxo-MEHP: 9.87 (8.72, 11.0) ∑(GM) = 26.5	
Urinary MEP (μg/L)	1,816	98.0	34.4 (32.8, 36.0)	159 (138, 183)	BE: *n *= 0 (0.0)	2009–2010	415	35.2 (31.2, 39.8)	151 (114, 207)
Urinary MBzP (μg/L)	1,816	95.2	7.1 (6.8, 7.5)	27.8 (25.2, 30.6)	BE: *n *= 0 (0.0)	2009–2010	415	11.6 (9.51, 14.1)	63.9 (47.4, 76.8)
Urinary MnBP (μg/L)	1,355	99.9	34.8 (33.5, 36.2)	95.5 (87.3, 104.5)	—	2009–2010	415	21.7 (19.0, 24.8)	83.8 (59.6, 121)
Urinary MiBP (μg/L)	1,355	99.8	45.4 (43.6, 47.3)	131 (117, 147)	—	2009–2010	415	10.2 (9.10, 11.4)	35.7 (28.8, 46.9)
Mothers
Mercury in hair (μg/g)	1,839	90.5	0.225 (0.216, 0.234)	1.200 (1.068, 1.349)	JECFA: *n *= 62 (3.4)	1999–2000	1,726	0.20 (0.16, 0.24)	1.11
Urinary cotinine (μg/L)	1,800	62.4	2.75 (2.41, 3.14)	1,182 (974, 1,434)	—	—	—	—	—
Urinary cadmium (μg/L)	1,685	93.8	0.219 (0.211, 0.228)	0.620 (0.580, 0.663)	HBM-I: *n *= 49 (2.9) HBM–II: *n *= 0 (0.0) BE: *n *= 26 (1.5)	2009–2010	1,450	0.188 (0.172, 0.206)	0.740 (0.620, 0.880)
Urinary DEHP metabolites (μg/L)^*e*^	1,800	81.6	29.2 (28.1, 30.3)	91 (84, 100)	HBM-I: *n *= 19 (1.0) BE: *n *= 28 (1.5)	2009–2010	1,350	MEHP: 1.39 (1.21, 1.60) 5OH-MEHP: 11.0 (9.58, 12.8) 5-oxo-MEHP: 7.09 (6.17, 8.14) ∑(GM) = 19.5	
Urinary MEP (μg/L)	1,800	95.2	48.2 (45.6, 51.0)	252 (221, 287)	BE: *n *= 0 (0.0)	2009–2010	1,350	67.8 (60.3, 76.4)	548 (392, 675)
Urinary MBzP (μg/L)	1,800	91.8	4.5 (4.3, 4.7)	17.7 (16.1, 19.5)	BE: *n *= 0 (0.0)	2009–2010	1,350	6.04 (5.38, 6.77)	29.3 (24.5, 36.9)
Urinary MnBP (μg/L)	1,347	99.4	23.9 (23.0, 24.9)	66.2 (60.5, 72.4)	—	2009–2010	1,350	14.7 (13.1, 16.5)	57.7 (52.7, 63.9)
Urinary MiBP (μg/L)	1,347	99.4	30.1 (28.9, 31.4)	88 (81, 96)	—	2009–2010	1,350	7.50 (6.68, 8.43)	29.1 (25.3, 33.5)
Abbreviations: BE, biomonitoring equivalent; DEHP, di(2-ethylhexyl)phthalate; ∑(GM), sum of geometric means of MEHP, 5OH-MEHP, and 5oxo-MEHP; HBM-I, human biomonitoring value I; HBM-II, human biomonitoring value II; JECFA, Joint FAO/WHO Expert Committee on Food Additives; LOQ, limit of quantification; MBzP, monobenzyl phthalate; MEP, monoethyl phthalate; MiBP, monoisobutyl phthalate; MnBP, mono-*n*-butyl phthalate; P90, 90th percentile. ^***a***^LOQs ranged from 0.001 to 0.137 μg/g for mercury in hair, 0.1–1.2 μg/L for urinary cotinine, 0.001–0.2 μg/L for urinary cadmium, 0.3–3.9 μg/L for urinary MEHP, 0.1–9.2 μg/L for urinary 5OH-MEHP, 0.1–6.2 μg/L for urinary 5oxo-MEHP, 0.5–11 μg/L for urinary MEP, 0.2–5 μg/L for urinary MBzP, 0.5–4.4 μg/L for urinary MnBP, and 0.5–4.9 μg/L for urinary MiBP. ^***b***^Geometric means and 90th percentiles are weighed but not adjusted for confounders (see “Methods”). ^***c***^Health-based exposure values are available for mercury: JECFA guideline = 2.3 μg/g (WHO 2004); cadmium: HBM-I in children = 0.5 μg/L; HBM-II in children = 1 μg/L; HBM-I in adults = 1.0 μg/L; HBM-II in adults = 4.0 μg/L (Schulz et al. 2012); BE in children and in mothers = 1.2 μg/L (Hays et al. 2008); phthalate metabolites: HBM-I value for DEHP metabolites are based on the sum of 5OH-MEHP and 5oxo-MEHP and equal 500 μg/L in children and 300 μg/L in adults (Schulz et al. 2012); BEs for DEHP metabolites are based on the sum of MEHP, 5OH–MEHP, and 5oxo-MEHP: 260 μg/L in both children and mothers (Aylward et al. 2009a); BE for MEP in mothers and children = 18 mg/L (Aylward et al. 2009b); BE for MBzP in children and adults = 3.8 mg/L (Aylward et al. 2009b). ^***d***^NHANES: data for urinary cadmium and urinary phthalate metabolites from *The Fourth National Report on Human Exposure to Environmental Chemicals, Updated Tables, March 2013* (Centers for Disease Control and Prevention 2013); data for mercury in hair from McDowell et al. (2004). Data for COPHES/DEMOCOPHES children are compared with NHANES subgroup “Age group 6–11 years”; data for COPHES/DEMOCOPHES mothers are compared with NHANES subgroup “Females.” ^***e***^Urinary DEHP metabolites: sum of MEHP, 5OH-MEHP, and 5oxo-MEHP.									

Cadmium levels in mothers were significantly higher in active smoking mothers and this was independent of age. The GMs were higher in mothers than in children (Table 2). Older mothers had 25% (95% CI: 18, 32%) higher levels than younger mothers (see Supplemental Material, Table S9). Levels in mothers and children showed a low but significant correlation (Spearman’s *r* = 0.24, *p* < 0.001, *n* = 1,660). After adjustment for age and smoking, mothers from families with a tertiary education had 34% (95% CI: 17, 54%) lower levels compared with those with a primary education. In children, except for age and creatinine, no significant determinants were identified (see Supplemental Material, Table S8).

The urinary levels of MEHP, 5OH-MEHP and 5oxo-MEH were highly correlated (Pearson’s *r* > 0.70), so their sum was used in the analyses. The GMs of urinary phthalate metabolites [except MEP, related to use of personal care products (PCPs)] were higher in children than in mothers (Table 2). Phthalate levels of mothers and children were significantly correlated (*p* < 0.001): Spearman’s *r* ranged between 0.40 and 0.49. Multiple regression models (Table 3) showed that younger children of 5–8 years showed higher levels compared with the older group of 9–11 years. Participants from families who reported having PVC (polyvinyl chloride) floors or walls had significantly increased levels of MBzP and MiBP in children and mothers and of MnBP in children (Tables 3 and 4). A small effect for MiBP was seen in mothers who reported renovation in the house in the previous 2 years. Frequent use of PCPs increased urinary MEP levels in mothers and children and urinary MiBP levels in children. Unexpectedly, urinary levels of di(2-ethylhexyl) phthalate (DEHP) metabolites and MnBP in mothers were lower in frequent PCP users. High consumption of ice cream was associated with higher urinary levels of DEHP metabolites and MBzP levels in children and with higher MnBP and MBzP levels in mothers. High consumption of chewing gum was related to higher urinary levels of DEHP metabolites in children and to higher MEP levels in mothers. After adjustment for confounders and significant covariates, educational level was still a predictor of phthalate biomarkers—that is, significantly higher urinary levels were found for DEHP metabolites in mothers from families with a primary education, for MiBP (mothers) and MEP (children) in families with secondary education, and for MnBP (children) in families with tertiary education.

**Table 3:** Determinants of exposure to urinary phthalate metabolites (μg/L): multiple regression models in children.

Parameters	Category	Estimate (95% CI) for change (multiplicative factor)				
		DEHP	MEP	MBzP	MnBP	MiBP
Age^*a*^	5–8 years	1.19 (1.11, 1.27)	1.15 (1.04, 1.26)	1.15 (1.06, 1.26)	1.15 (1.07, 1.24)	1.19 (1.10, 1.28)
	9–11 years	1.00	1.00	1.00	1.00	1.00
Sex^*a*^	Boys	NS	NS	NS	0.91 (0.85, 0.98)	0.92 (0.85, 0.99)
	Girls				1.00	1.00
Urinary creatinine level^*a*^	300–900 mg/L	0.46 (0.42, 0.51)	0.41 (0.36, 0.47)	0.41 (0.37, 0.47)	0.45 (0.41, 0.50)	0.45 (0.40, 0.50)
	900–1,500 mg/L	0.75 (0.69, 0.83)	0.68 (0.61, 0.77)	0.69 (0.62, 0.78)	0.73 (0.66, 0.81)	0.72 (0.65, 0.80)
	1,500–3,000 mg/L	1.00	1.00	1.00	1.00	1.00
Urine sampling period	< 10 hr	NS	1.20 (1.06, 1.35)	NS	NS	NS
	10–11 hr		1.14 (1.02, 1.29)			
	≥ 11 hr		1.00			
Morning urine	Yes	NS	NS	1.98 (1.17, 3.36)	NS	NS
	No			1.00		
Educational level of the family	Primary	NS	0.91 (0.81, 1.03)	NS	0.91 (0.81, 1.03)	NS
	Secondary		0.89 (0.82, 0.97)		0.89 (0.82, 0.97)	
	Tertiary		1.00		1.00	
Use of personal care products^*b*^	Moderate to high use	NS	1.24 (1.13, 1.37)	NS	NS	1.13 (1.03, 1.23)
	Low use		1.00			1.00
Ice cream consumption	Several times/week	1.12 (1.01, 1.25)	NS	1.18 (1.02, 1.36)	NS	NS
	Once/week or less	1.00		1.00		
Gum consumption	Several times/week	1.10 (1.02, 1.18)	NS	NS	NS	NS
	Once/week or less	1.00				
PVC in floors/walls	Yes	NS	NS	1.50 (1.34, 1.68)	1.19 (1.08, 1.32)	1.22 (1.09, 1.35)
	No			1.00	1.00	1.00
NS, not significant. For phthalate abbreviations, see Table 2. ^***a***^The confounders urinary creatinine level, sex, and age were forced in the multiple regression models, even if not significant. ^***b***^Use of personal care products (PCPs) is calculated as a score based on the frequency (never to daily) of nine PCP groups (makeup, eye makeup, shampoo, hair-styling products, body lotions and creams, fragrances, deodorant, massage oil, and nail polish).						

**Table 4:** Determinants of exposure to urinary phthalate metabolites (μg/L): multiple regression models in mothers.

Parameters	Category	Estimate (95% CI) for change (multiplicative factor)				
		DEHP	MEP	MBzP	MnBP	MiBP
Age^*a*^	≤ 35 years	NS	NS	NS	0.81 (0.73, 0.89)	NS
	35–40 years				0.93 (0.86, 1.01)	
	> 40 years				1.00	
Body mass index	Normal Weight	NS	NS	NS	1.15 (1.02, 1.29)	NS
	Overweight				1.09 (0.96, 1.24)	
	Obese				1.00	
Urinary creatinine level^*a*^	300–900 mg/L	0.35 (0.32, 0.38)	0.32 (0.28, 0.37)	0.33 (0.30, 0.37)	0.35 (0.32, 0.38)	0.38 (0.35, 0.41)
	900–1,500 mg/L	0.62 (0.57, 0.68)	0.63 (0.55, 0.72)	0.59 (0.54, 0.65)	0.60 (0.55, 0.66)	0.61 (0.56, 0.67)
	1,500–3,000 mg/L	1.00	1.00	1.00	1.00	1.00
Urine sampling period	< 7 hr	0.87 (0.79, 0.97)	NS	NS	NS	NS
	7–9 hr	0.97 (0.88, 1.06)				
	≥ 9 hr	1.00				
Educational level of the family	Primary	1.20 (1.05, 1.37)	NS	NS	NS	1.09 (0.97, 1.23)
	Secondary	1.04 (0.96, 1.13)				1.11 (1.02, 1.21)
	Tertiary	1.00				1.00
Use of personal care products^*b*^	High use	0.91 (0.84, 0.98)	1.40 (1.25, 1.56)	NS	0.92 (0.86, 0.99)	NS
	Moderate to low use	1.00	1.00		1.00	
Ice cream consumption	Several times/month	NS	NS	1.13 (1.03, 1.24)	1.10 (1.01, 1.19)	NS
	Once/month or less			1.00	1.00	
Gum consumption	Several times/week	NS	1.19 (1.06, 1.34)	NS	NS	NS
	Once/week or less		1.00			
PVC in floors/walls	Yes	NS	NS	1.32 (1.19, 1.47)	NS	1.15 (1.04, 1.26)
	No			1.00		1.00
Renovation in house	Yes	NS	NS	NS	NS	1.08 (1.00, 1.16)
	No					1.00
NS, not significant. For phthalate abbreviations, see Table 2. ^***a***^The confounders urinary creatinine level and age were forced in the multiple regression models, even if not significant. ^***b***^Use of personal care products (PCPs) is calculated as a score based on the frequency (never to daily) of nine PCP groups (makeup, eye makeup, shampoo, hair-styling products, body lotions and creams, fragrances, deodorant, massage oil, and nail polish).						

In mothers, the effect of active smoking on cotinine levels was dominant (see Supplemental Material, Table S7). Levels in mothers and children correlated strongly (Spearman’s *r* = 0.71, *p* < 0.001, *n* = 1,777). The younger children of 5–8 years showed 16% (8, 25%) higher levels compared to the older group of 9–11 years (see Supplemental Material, Table S6). In children, environmental tobacco smoke (ETS) at home was the strongest predictor. Compared with children who were never exposed to ETS at home, children with daily exposure had five times higher values [504% (95% CI: 429, 593%)], and children with less than daily exposure had almost double values [181% (95% CI: 155, 211%)]. Exposure to ETS in other places than home resulted in 19% (95% CI: 10, 29%) higher values. Compared with children from families with a tertiary education, those with a secondary education had 20% (95% CI: 10, 30%) higher cotinine levels in urine, and those with primary education had 49% (95% CI: 29, 72%) higher values.

*The geographical aspect*. Residence in urban or rural area was not a significant determinant of internal exposure at the European Union level. Only mercury in hair showed, independently of fish consumption, higher levels in urban areas compared with rural areas: 35% (95% CI: 23, 47%) higher in children and 30% (95% CI: 19, 41%) in mothers (see Supplemental Material, Tables S4 and S5).

The average biomarker concentrations varied significantly among the European countries. This holds for the unadjusted data (see Supplemental Material, Tables S20–S35) and for data after adjustment for age, sex, and weighting for equal group sizes (Figure 1). The average biomarker concentrations of mercury in hair of Spanish and Portuguese children were, respectively, six and seven times higher than the European average. Cadmium varied less among the countries: Average urinary cadmium levels in Polish and Slovak mothers were respectively 1.9 and 1.7 times higher than the European average. In Romania and Hungary, average cotinine levels were, respectively, 2.4 and 2.2 times higher than the European average, reflecting the weak antismoking legislation in these countries. Swedish children had, on average, three times higher urinary MBzP levels than the average European value. Slovak children had almost twice the average European biomarker concentrations of DEHP metabolites, and Polish children showed the highest average levels of MnBP and MiBP. Average MEP levels in Spain were six times higher than the European average. The heat map (Figure 2) shows that biomarker data from mothers and children clustered together except in the Czech Republic and the Slovak Republic. Overall the biomarker clustering followed geographical grouping. The Southern European countries (Spain, Portugal) clustered separately from the other countries; Eastern European countries (Romania, Hungary, Poland, the Czech Republic, and the Slovak Republic) formed a further cluster; Western European countries (Germany, Belgium, Luxembourg, and Denmark) also showed fairly good resemblance.

Although the sampling frame of the European biomonitoring program differs from that of the U.S. national program, the geometric means and P90 of COPHES/DEMOCOPHES are well in line with the results obtained in NHANES (Centers for Disease Control and Prevention 2013; McDowell et al. 2004) (Table 2). For MiBP, higher values were observed in Europe compared with the United States (factor 3–4), in both mothers and children (Table 2). Differences for other biomarkers were modest, with a trend in Europe for lower biomarker concentrations of MBzP and MEP, higher concentrations of MnBP and DEHP, and similar levels for cadmium and mercury.

Available health-based guidance values allow us to put the observed biomarker concentrations in a risk context. Few participants exceeded these values (Table 2). The P90 of the biomarker values is far below the guidance values; only the urinary cadmium P90 of mothers and children was within a factor 2 of the concentration below which no risk for adverse health effects is expected (Schulz et al. 2012), and for mercury they are below a factor 3.

## Discussion

This first Europe-wide program provides biomarker data from mothers and children of 17 European countries. Because we recruited in one rural and one urban area per country, our sample was not representative for the European Union population. Yet the recruited sample had a smoking behavior similar to that of the average European population (Currie et al. 2012). Also, the countries ranked in their reported fish consumption here according to national statistics (Food and Agriculture Organization 2013). The educational level of the participants was skewed toward a higher educational level. The study design allowed us to conclude that exposure to mercury, cadmium, phthalates, and nicotine is widespread in the European population.

Differences in environment and lifestyle influenced individual biomarker values and country-specific averages. If we compared average levels among countries, the biomarker patterns varied according to geographic trends. Yet few study participants exceeded the available health-based guidance values. The major strength of our study is the comparable data from 17 European countries produced through a harmonized process, including the use of a commonly developed protocol, intensive training and capacity building for field work, chemical analyses, reporting and communication, as well as stringent quality control programs for chemical and data analysis. This allowed us to measure both well-known pollutants such as cadmium, cotinine, or mercury and emerging chemicals such as phthalates.

Our study identified younger children as more exposed than older children to phthalates (except MEP), cotinine, and mercury. These results are in line with U.S. data for exposure to phthalates (Silva et al. 2004) and ETS (Bernert et al. 2010). The underlying reasons cannot be derived from this study but may be explained by higher exposure relative to body size through inhalation of dust or food intake; by typical exposure patterns in children, such as contact with toys, more time spent on the floor, and more frequent hand-to-mouth contact; or by differences in metabolism. Additionally, the higher cotinine levels in younger children might be attributable to the fact that they spend more time at home, and thus may be more exposed to nicotine, since smoking in public buildings is much more controlled than in private homes. We observed a significant influence of social class (represented by the highest educational level within the family) on each of the biomarker levels even after adjustment for confounders and significant covariates: Mercury level in hair increased in children and mothers if social class was higher, whereas cotinine, cadmium, and phthalate metabolites were lower with increasing educational level of the family. Perhaps underlying lifestyle factors that vary with socioeconomic status and that were not considered in the questionnaires may account for these findings. These associations between social class and biomarker concentrations are in line with U.S. data (Tyrrell et al. 2013) and may be mediated partly by smoking, occupation, and diet (fish consumption, local food, convenience food). Our findings thus indicate that public health remediation measures to decrease environmental exposure and disease burden within a society should be stratified according to age groups and social strata within the population.

Fish consumption and social status were identified as important and independent determinants of mercury levels, both in mothers and children. This is in line with results from several populations with moderate to high fish consumption (Deroma et al. 2013). Mercury levels in children and in women of childbearing age are important parameters to monitor because pre- and postnatal mercury exposure, even at low levels, has adverse neurodevelopmental effects (Karagas et al. 2012). Although several high fish–consuming countries such as France, Finland, Lithuania, Malta, and Italy are not participating in DEMOCOPHES at present, 1.4% of the children and 3.4% of the mothers in our study population had mercury levels above the JECFA/WHO provisional threshold value of 2.3 μg/g hair (WHO 2004). This proportion differs considerably by country, with 0% of participants exceeding the threshold in most Northern and Central European countries and up to 33% of the mothers with levels above the safe dose in countries with high fish consumption, with implications for loss of IQ points and costs (Bellanger et al. 2013). If these data urge policy makers to take actions, current biomarker concentrations can be used as baseline for follow-up, both for the exposure of the population and the environment. The major exposure route for DEHP is food (Koch and Calafat 2009). Therefore, we were not surprised to find an association between DEHP metabolites with chewing gum and ice cream consumption. Most probably, these two food items are not specific sources, but rather represent predilection for flavored, packaged, or processed food, and thus may be proxies for convenience food. The association between urinary MBzP and PVC materials in the home is in accordance with recent findings in children (Carlstedt et al. 2012). Although high-molecular-weight phthalates such as DEHP are the major phthalates used in PVC, no association was found between the presence of PVC at home and urinary DEHP metabolites. Given that DEHP exposure is dominated by foods (Koch et al. 2013) and that DEHP house dust does not correlate with DEHP body burden (Becker et al. 2004), a significant correlation was not really expected. The lower levels of DEHP metabolites and MnBP in mothers who were high PCP users were not expected and may relate to cross-correlation with other personal habits. The relative levels of phthalate metabolites differ substantially among countries, which points to different sources, products on the market, or behavior characteristics. Despite legal restrictions on the use of DEHP, di-*n*-butyl phthalate, and diisobutyl phthalate as imposed by European Union directives, these compounds are still ubiquitous in Europeans. They are short-lived in the body, implying that exposures to these compounds are still part of current daily life. Diethyl phthalate, one of the principal phthalates in cosmetic products (Koch and Calafat 2009), is not yet restricted. High levels of its metabolite MEP were found.

The health impact of cigarette smoking is well documuented (U.S. Department of Health and Human Services 2004). The home environment appears to be the most important predictor of the cotinine levels in children. Further awareness of parents therefore is needed. The importance of anti-smoking legislation pays off, as countries with stronger legislation that has been longer in place showed the lowest cotinine levels (European Union 2011). The effectiveness of anti-smoking legislation on health outcomes has been demonstrated on a population level (Cox et al. 2013).

## Conclusion

This HBM study presents the first steps, for Europe as a whole, to register internal chemical exposures at the individual level. Although the sampling protocol is not yet representative for the geographical distribution of the population in the country, the results show remarkable differences in the biomarker concentration profiles by country residence. Personal habits and lifestyle are strong determinants of internal exposure. The harmonized protocols and stringent quality control measures ensure that these are true differences, not related to variability in protocols, analytical measurements, or interpretation. These data offer policy makers direct means by which to evaluate whether implementation of protective measures and legislation related to chemicals are adequate to protect the health of the entire population or whether they need to be adjusted.

## Supplemental Material

(3.4 MB) PDF
