# Supplementary material for: First Steps toward Harmonized Human Biomonitoring in Europe: Demonstration Project to Perform Human Biomonitoring on a European Scale
Source: Environ Health Perspect. 2014 Dec 11;123(3):255–63. doi: 10.1289/ehp.1408616 (PMC4348748; doi:10.1289/ehp.1408616)
Supplement: (3.4 MB) PDF [file ehp.1408616.s001.508.pdf]

Environmental Health Perspectives doi:10.1289/ehp.1408616

Supplemental Material

First Steps toward Harmonized Human Biomonitoring in Europe: Demonstration Project to Perform Human Biomonitoring on a European Scale

Elly Den Hond, Eva Govarts, Hanny Willems, Roel Smolders, Ludwine Casteleyn, Marike Kolossa-Gehring, Gerda Schwedler, Margarete Seiwert, Ulrike Fiddicke, Argelia Castaño, Marta Esteban, Jürgen Angerer, Holger M. Koch, Birgit K. Schindler, Ovnair Sepai, Karen Exley, Louis Bloemen, Milena Horvat, Lisbeth E. Knudsen, Anke Joas, Reinhard Joas, Pierre Biot, Dominique Aerts, Gudrun Koppen, Andromachi Katsonouri, Adamos Hadjipanayis, Andrea Krskova, Marek Maly, Thit A. Mørck, Peter Rudnai, Szilvia Kozepesy, Maurice Mulcahy, Rory Mannion, Arno C. Gutleb, Marc E. Fischer, Danuta Ligocka, Marek Jakubowski, M. Fátima Reis, Sónia Namorado, Anca Elena Gurzau, Ioana-Rodica Lupsa, Katarina Halzlova, Michal Jajcaj, Darja Mazej, Janja Snoj Tratnik, Ana López, Estrella Lopez, Marika Berglund, Kristin Larsson, Andrea Lehmann, Pierre Crettaz, and Greet Schoeters

Details on Statistical Analysis

Methods

Study protocol

The list of ethics committees is given in Supplemental Material, Table S1.

Table S1. Overview of ethics committee per country.

Country	Ethics committee
BE - Belgium	Ethics Committee of the University of Antwerp
CH - Switzerland	Kantonale Ethikkomission Bern (KEK)
CY - Cyprus	Cyprus National Bioethics Committee (CNBC).
CZ - Czech Republic	Ethical Committee of the NIPH
DE - Germany	Ethics Committee of the Ruhr-University, Bochum
DK - Denmark	De Videnskabsetiske Komiteer for Region Hovedstaden
ES - Spain	Comité de Ética de la Investigación y Bienestar Animal
HU - Hungary	Medical Research Counsil, Scientific and Ethical Committee
IE- Ireland	Royal College of Surgeons Research and Ethics Commitee
LU - Luxembourg	Comité d'Ethique de Recherche (CNER)
PL - Poland	Bioethical Committee at the Nofer Institute of Occupational Medicine in Lodz
PT - Portugal	Comissão de Ética Centro Hospitalar Lisboa Norte / Faculdade de Medicina de Lisboa
RO - Romania	Ethics Committee of the Environmental Health Center (EHC) for the Institutional Review Board (IRB)
SE - Sweden	Regionala Etixprövningsnämnden (The regional ethical review board), Stockholm
SI - Slovenia	Komisija Republike Slovenije za medicinsko etiko / National Medical Ethics Comittee
SK - Slovac Republic	Ethical Committee of the Health Authority of the Slovak Republic
UK - United Kingdom	Riverside Research Ethic Committee, NHS Brighton & Hove.

Identification of determinants of exposure

For each biomarker of exposure the relation with possible determining factors was studied by univariate and multiple regression techniques. All analyses were done separately for the mothers and the children.

Confounders and covariates are listed in Supplemental Material, Table S1. Confounders are *a priori* defined variables that are known to be related to the biomarker. Covariates are possible determinants; its relationship with the biomarker is tested within the study group. All confounders and covariates were put in the models as categorical variables.

Both for univariate and for mutiple models, linear mixed models were used instead of the ordinary linear regression models. Hence, the clustered design was taken into account in mixed effect analysis. Within a country, participants were recruited in a similar way, and thus the mothers (or children) within one country may be considered as dependent measures. Mothers (or children) from one country may have 'more identical' biomarker values than mothers (or children) from another country. This dependency between the biomarker values could be introduced into a model by a random effect. The correlation between the biomarker values within a country was estimated by the model. The intraclass correlation coefficient gives the proportion of variability in the biomarker values due to the variability between countries. The introduction of the random effects into the model will change the confidence intervals of the estimates. Explanatory variables of interest were included in the model as fixed effects.

First, univariate models were developed for all covariates. In a second step, multiple regression models were built including those determining factors which are significant at the 0.25

significance level in the univariate analyses. The confounders are fixed into the model. Important determining factors were identified by stepwise selection procedures (this is a combination of forward and backward selection procedures) in which we set $p<0.05$ to stay in the model. As such a final linear mixed model is obtained.

Quantitative relationships between the covariates and the biomarkers were calculated from the estimates of the linear mixed model, assuming that, when quantifying the relation of one covariate with the biomarker, all other covariates in the model are fixed at the population mean.

The problem of multicollinearity, that is the existence of a high degree of linear correlation amongst two or more explanatory variables in a regression model (Neter et al. 1996) was examined. Multicollinearity makes it difficult to separate the effects of the explanatory variables on the dependent variable. In the presence of multicollinearity, the estimate of one variable's impact on Y while controlling for the others tend to be less precise than if the predictors were uncorrelated with one another. Spearman correlation coefficients between the different explanatory variables were calculated; highly correlated variables were not included in the same model. The effects of multicollinearity were analyzed using variance inflation factors. If the variance inflation factor was larger than 10 then multicollinearity was concluded (Fox 1991).

Table S2. List of confounders (Conf) and covariates (Cov) to be examined in relation with biomarkers of exposure.

Subgroups/strata	Mercury in hair (µg/g)	Urinary cadmium (µg/L)	Urinary cotinine (µg/L)	Urinary phthalate metabolites (µg/L)
Creatinine	NA	Conf	Conf	Conf
Urinary volume	NA	Cov	Cov	Cov
Time period of urine collection	NA	Cov	Cov	Cov
Morning urine	NA	Cov	Cov	Cov
Age	Conf	Conf	Conf	Conf
Body-mass index	NA	NA	NA	Cov
Smoking	NA	Conf[a]	Cov	NA
ETS	NA	Cov	Cov	NA
Food consumption (specific items)	Cov	Cov	NA	Cov
Water consumption	Cov	Cov	NA	Cov
Neighborhood of industry (spec.)	Cov	Cov	NA	Cov
Fuel sources	NA	Cov	NA	NA
House renovation /redecoration	NA	Cov	NA	Cov
PVC in house	NA	NA	NA	Cov
Traffic exposure	NA	Cov	NA	Cov
Soldering	Cov	Cov	NA	NA
Amalgam fillings	Cov	NA	NA	NA
Skin bleaching	Cov	NA	NA	NA
Broken mercury thermometer	Cov	NA	NA	NA
Broken energy-saving lamps	Cov	NA	NA	NA
Hair treatment	Cov	NA	NA	NA
Use of personal care products	NA	NA	NA	Cov
Educational level	Cov	Cov	Cov	Cov
Rural / urban	Cov	Cov	Cov	Cov
Gender	Conf	Conf	Conf	Conf
Contact with toys	NA	NA	NA	Cov
Use of gloves	NA	NA	NA	Cov

Conf: confounder; Cov: covariate; NA: not applicable; ETS: environmental tobacco smoke; PVC: polyvinyl chloride

[a]Initially, smoking was a confounder in the models for Cd in children but this variable was not retained as confounder since there were no smokers in the children's study group.

The assumption of normality was checked with informal diagnostic residual plots and the Kolmogorov–Smirnov test (Neter et al. 1996). Influence diagnostics (e.g. restricted likelihood distance, Cook's D, CovRatio) were used to quantify the influence of one or more observations by computing parameters estimates based on all data points, removing the cases in question from the data, refitting the model, and computing statistics based on the change between full-data and reduced-data estimates.

The models to explore the relation between possible determining factors were built on the central database, without any weighing. As a sensitivity analysis weights were added to the final model in such a way that each country contributed equally (except Luxembourg and Cyprus for half) to the model. Results with and without weighing were compared.

Comparison of results between countries

European exposure values were calculated as geometric mean (with 95% confidence interval). These exposure values were calculated separately for mothers and children. For urinary markers, exposure values were calculated in µg/L and in µg/g creatinine. Exposure values were weighted so that all countries contribute equally to the exposure value. All countries were assigned a weight as such that they contribute for 120 individuals to the exposure value, only Luxembourg and Cyprus contributed for half (60 individuals). Exposure values were both calculated on the raw data as adjusted for the specific confounders of the biomarker (Supplemental Material, Table S1).

The level of the biomarkers in each country was compared with the European exposure values. Hence, it is tested whether mean levels of a biomarker in a country were significantly above or below the pilot reference mean at the 5% significance level, both for the unadjusted values as

after adjustment for confounders. The natural logarithm of the biomarker values was used for the statistical analyses. In order to test the overall differences between the 17 countries, the significance of country was tested in a multiple regression models (based on a F-test), either with or without inclusion of the confounders in the model. If the null hypothesis was not rejected (i.e. there were no significant differences between the countries at the 5% significance level), no further testing was done. If the null hypothesis was confirmed, further testing was done by comparing the mean in each country with the pilot reference mean. The significance of these differences was determined by multiple regression models, either with or without inclusion of confounders.

References

Fox, J. 1991. Regression Diagnostics: an Introduction. Ed. Sage. CA.

Neter J, Kutner M, NachtsheimC, Wasserman W. 1996. Applied Linear Statistical Models. Ed. McGraw-Hill/Irwin. New York: (4th edition).

1. Descriptive statistics

The descriptive statistics for all biomarkers are presented in Supplemental Material, Table S3. The data are given in µg/g hair for mercury and both in µg/L and µg/g creatinine for urinary markers, separately for children and mothers.

Geometric means and percentiles are calculated on the basis of the ‘raw’ data, i.e. without weighing or correction for clustering, and without adjustment for confounders or covariates.

Table S3. Descriptive statistics for biomarkers of exposure in 17 European countries, separately for mothers and children.

Biomarker	N	% >LOQ	GM (95%CI)	min.	P10	P25	P50	P75	P90	P95	max.
**Children**											
Mercury in hair (µg/g)	1836	85.9	0.143 (0.135, 0.152)	0.005	0.030	0.065	0.144	0.352	0.818	1.289	7.100
Cadmium in urine (µg/L)	1698	70.1	0.067 (0.064, 0.071)	0.005	0.020	0.032	0.080	0.131	0.220	0.270	0.640
Cadmium in urine (µg/g crt)	1698	70.1	0.065 (0.062, 0.068)	0.003	0.017	0.038	0.075	0.123	0.181	0.233	1.037
Cotinine in urine (µg/L)	1818	57.6	0.82 (0.77, 0.87)	0.05	0.24	0.35	0.60	1.53	5.10	11.00	269.8
Cotinine in urine (µg/g crt)	1818	57.6	0.79 (0.74, 0.83)	0.03	0.19	0.32	0.62	1.61	4.98	10.24	152
DEHP metabolites in urine (µg/L)	1816	85.6	48.6 (46.8, 50.5)	1.84	17.0	28.4	46.8	80.9	141.0	213.4	1356
DEHP metabolites in urine (µg/g crt)	1816	85.6	46.8 (45.2, 48.5)	2.04	18.7	28.7	45.5	73.1	120.3	170.1	3616
MEP in urine (µg/L)	1816	98.0	35.8 (34.0, 37.7)	0.71	9.0	16.4	33.0	69.5	160.0	254.3	6858
MEP in urine (µg/g crt)	1816	98.0	34.4 (32.7, 36.2)	0.94	9.9	16.7	29.3	65.7	141.8	242.4	4017
MBzP in urine (µg/L)	1816	95.2	7.4 (7.0, 7.7)	0.1	2.3	3.5	7.0	14.0	27.8	40.1	929
MBzP in urine (µg/g crt)	1816	95.2	7.1 (6.8, 7.4)	0.18	2.1	3.5	6.6	12.9	26.0	42.2	603
MnBP in urine (µg/L)	1355	99.9	35.8 (34.3, 37.4)	2.0	12.5	21.0	36.0	60.3	98.0	139.0	865
MnBP in urine (µg/g crt)	1355	99.9	34.6 (33.2, 36.0)	0.83	14.2	21.3	33.9	54.7	88.1	131.3	883
MiBP in urine (µg/L)	1355	99.8	47.5 (45.3, 49.7)	2.3	16.0	26.0	47.9	83.0	135.0	199.9	1940
MiBP in urine (µg/g crt)	1355	99.8	45.9 (43.9, 47.9)	1.7	17.0	27.6	44.8	75.2	125.9	167.6	1634
**Mothers**											
Mercury in hair (µg/g)	1839	90.5	0.230 (0.217, 0.244)	0.005	0.051	0.099	0.231	0.538	1.28	1.89	9.66
Cadmium in urine (µg/L)	1685	93.8	0.218 (0.209, 0.227)	0.005	0.076	0.130	0.230	0.390	0.624	0.820	2.780
Cadmium in urine (µg/g crt)	1685	93.8	0.193 (0.187, 0.200)	0.003	0.077	0.128	0.200	0.311	0.467	0.592	2.459
Cotinine in urine (µg/L)	1800	62.4	2.95 (2.56, 3.40)	0.05	0.24	0.35	0.80	4.90	1237	1861	4991
Cotinine in urine (µg/g crt)	1800	62.4	2.62 (2.27, 3.02)	0.02	0.19	0.33	0.74	4.28	927	1591	5145
DEHP metabolites in urine (µg/L)	1800	84.3	30.2 (29.0, 31.5)	1.87	9.7	16.3	29.8	53.2	93.0	132.0	2721
DEHP metabolites in urine (µg/g crt)	1800	84.3	26.8 (25.9, 27.8)	1.80	10.7	16.5	25.5	41.7	70.2	104.3	1540
MEP in urine (µg/L)	1800	98.3	49.6 (46.7, 52.6)	1.3	9.8	19.5	46.0	114.6	259	448	6761
MEP in urine (µg/g crt)	1800	98.3	44.0 (41.7, 46.5)	1.26	10.3	18.2	40.7	92.1	209	342	3259
MBzP in urine (µg/L)	1800	91.8	4.7 (4.5, 4.9)	0.1	1.4	2.5	4.5	8.7	18.0	25.9	698
MBzP in urine (µg/g crt)	1800	91.8	4.2 (4.0, 4.4)	0.128	1.4	2.3	4.0	7.0	13.7	20.8	433
MnBP in urine (µg/L)	1347	99.4	25.0 (23.9-26.1)	1.68	8.9	14.9	24.5	41.4	68.0	97.0	2800
MnBP in urine (µg/g crt)	1347	99.4	22.3 (21.5, 23.2)	0.88	9.8	14.7	22.0	33.9	51.6	67.6	2059
MiBP in urine (µg/L)	1347	99.4	31.6 (30.3-33.1)	2.45	11.0	18.0	31.0	56.0	89.0	124	702
MiBP in urine (µg/g crt)	1347	99.4	28.3 (27.2, 29.4)	0.98	12.7	17.8	27.7	44.8	69.4	94.3	347

N: number; LOQ: limit of quantification; GM: geometric mean; 95% CI: 95% confidence interval; min.: minimum; P: percentile; max.: maximum; DEHP: di(2-ethylhexyl)phthalate; MEP: mono-ethyl phthalate; MBzP: mono-benzyl phthalate; MnBP: mono-n-butyl phthalate; MiBP: mono-iso-butyl phthalate; DEHP metabolites: sum of MEHP, 5OH-MEHP and 5oxo-MEHP.

Geometric means and percentiles are calculated on the basis of the 'raw' data, i.e. without weighing or correction for clustering, and without adjustment for confounders or covariates.

2. Identification of determinants of exposure

The results of the multiple regression models are given in Supplemental Material, Table S4 to Table S19.

Table S4. Determinants of exposure to mercury: multiple regression model in children.

Parameters	Estimate (95%CI) for change (multiplicative factor)	*p*-value	Overall *p*-value
**Gender**			
Boys	0.96 (0.89, 1.05)	0.37	0.37
Girls	1.00		
**Age**			
5-8 years	1.08 (1.00, 1.17)	0.06	0.06
9-11 years	1.00		
**Consumption of sea fish**			
Several times per week	1.46 (1.26, 1.69)	<0.001	<0.001
Once per week or less	1.00		
**Consumption of shellfish**			
Several times per week	1.56 (1.35, 1.79)	<0.001	<0.001
Once per week or less	1.00		
**Consumption of fresh water fish**			
Several times per week	1.23 (1.08, 1.39)	<0.001	<0.001
Once per week or less	1.00		
**Educational level of the family**			
Primary	0.81 (0.69, 0.96)	0.01	<0.001
Secondary	0.81 (0.74, 0.90)	<0.001	
Tertiary	1.00		
**Area of residence**			
Urban	1.35 (1.23, 1.47)	<0.001	<0.001
Rural	1.00		

Number of observations in model: n=1798. Cluster variance: 0.70 (p=0.003); residual variance: 0.77; intra-class correlation coefficient: 0.47.

Table S5. Determinants of exposure to mercury: multiple regression model in mothers.

**Parameters**	**Estimate (95%CI) for change (multiplicative factor)**	***p*-value**	**Overall *p*-value**
**Age**			
≤ 35 years	0.85 (0.76, 0.95)	0.004	0.007
35-40 years	0.98 (0.89, 1.08)	0.69	
> 40 years	1.00		
**Consumption of sea fish**			
Several times per week	1.51 (1.34, 1.71)	<0.001	<0.001
Once per week or less	1.00		
**Consumption of sea food products**			
Several times per month	1.16 (1.00, 1.35)	0.047	0.047
Once per month or less	1.00		
**Consumption of shellfish**			
Several times per week	1.38 (1.24, 1.55)	<0.001	<0.001
Once per week or less	1.00		
**Consumption of fresh water fish**			
Several times per week	1.23 (1.11, 1.37)	<0.001	<0.001
Once per week or less	1.00		
**Educational level of the familiy**			
Primary	0.75 (0.64, 0.87)	<0.001	<0.001
Secondary	0.79 (0.72, 0.87)	<0.001	
Tertiary	1.00		
**Area of residence**			
Urban	1.30 (1.19, 1.41)	<0.001	<0.001
Rural	1.00		

Number of observations in model: n=1800. Cluster variance: 0.63 (p=0.003); residual variance: 0.69; intra-class correlation coefficient: 0.48.

Table S6. Determinants of urinary cotinine (µg/L): multiple regression model in children.

**Parameters**	**Estimate (95%CI) for change (multiplicative factor)**	***p*-value**	**Overall *p*-value**
**Urinary creatinine level**			
300-900 mg/L	0.89 (0.80 – 0.99)	0.03	0.10
900-1500 mg/L	0.92 (0.83, 1.01)	0.09	
1500-3000 mg/L	1.00		
**Gender**			
Boys	1.03 (0.96, 1.11)	0.42	0.42
Girls	1.00		
**Age**			
5-8 years	1.16 (1.08, 1.25)	<0.001	<0.001
9-11 years	1.00		
**ETS at home**			
Daily	5.04 (4.29, 5.93)	<0.001	<0.001
Less than daily	1.81 (1.55, 2.11)	<0.001	
Never	1.00		
**ETS elsewhere**			
Yes	1.19 (1.10, 1.29)	<0.001	<0.001
No	1.00		
**ETS in last 24 hours**			
Yes	2.64 (2.30, 3.04)	<0.001	<0.001
No	1.00		
**Educational level of the family**			
Primary	1.49 (1.29, 1.72)	<0.001	<0.001
Secondary	1.20 (1.10, 1.30)	<0.001	
Tertiary	1.00		

Number of observations in model: n=1809. Cluster variance: 0.25 (p=0.003); residual variance: 0.63; intra-class correlation coefficient: 0.28.

Table S7. Determinants of urinary cotinine (μg/L): multiple regression model in mothers.

Parameters	Estimate (95%CI) for change (multiplicative factor)	*p*-value	Overall *p*-value
**Urinary creatinine level**			
300-900 mg/L	0.90 (0.73, 1.11)	0.31	0.51
900-1500 mg/L	0.99 (0.82, 1.21)	0.97	
1500-3000 mg/L	1.00		
**Age**			
≤ 35 years	1.17 (0.93, 1.46)	0.17	0.22
35-40 years	1.17 (0.97, 1.42)	0.10	
> 40 years	1.00		
**Smoking + ETS in last 24 hours**			
Smoker	431 (350, 531)	<0.001	<0.001
Non-smoker, ETS in last 24 hrs	4.29 (3.21, 5.75)		
Non-smoker, no ETS in last 24 hrs	1.00		
**Educational level of the familiy**			
Primary	1.82 (1.34, 2.47)	<0.001	<0.001
Secondary	1.52 (1.27, 1.83)	<0.001	
Tertiary	1.00		

Number of observations in model: n=1794. Cluster variance: 0.18 (p=0.01); residual variance: 2.95; intraclass coefficient: 0.06.

Table S8. Determinants of urinary cadmium (µg/L): multiple regression model in children.

Parameters	Estimate (95%CI) for change (multiplicative factor)	*p*-value	Overall *p*-value
**Urinary creatinine level**			
300-900 mg/L	0.50 (0.46, 0.55)	<0.001	<0.001
900-1500 mg/L	0.68 (0.62, 0.74)	<0.001	
1500-3000 mg/L	1.00		
**Gender**			
Boys	0.98 (0.92, 1.04)	0.49	0.49
Girls	1.00		
**Age**			
5-8 years	0.93 (0.87, 0.99)	0.03	0.03
9-11 years	1.00		

Number of observations in model: n=1697. Cluster variance: 0.38 (p=0.003); residual variance: 0.4664; intra-class correlation coefficient: 0.45.

Table S9. Determinants of urinary cadmium (µg/L): multiple regression model in mothers.

Parameters	Estimate (95%CI) for change (multiplicative factor)	*p*-value	Overall *p*-value
**Urinary creatinine level**			
300-900 mg/L	0.35 (0.32, 0.38)	<0.001	<0.001
900-1500 mg/L	0.63 (0.58, 0.68)	<0.001	
1500-3000 mg/L	1.00		
**Age**			
≤ 35 years	0.75 (0.68, 0.82)	<0.001	<0.001
35-40 years	0.86 (0.79, 0.92)	<0.001	
> 40 years	1.00		
**Smoking + ETS at home**			
Smoker	1.31 (1.21, 1.43)	<0.001	<0.001
Non-smoker, ETS at home	1.10 (0.98, 1.24)	0.11	
Non-smoker, no ETS at home	1.00		
**Water consumption**			
Public water	1.28 (1.09, 1.51)	0.002	0.001
Commercial products	1.14 (0.95, 1.37)	0.17	
Well/private water	1.00		
**Educational level of the family**			
Primary	1.34 (1.17, 1.54)	<0.001	<0.001
Secondary	1.12 (1.04, 1.21)	0.003	
Tertiary	1.00		

Number of observations in model: n=1678. Cluster variance: 0.07 (p=0.005); residual variance: 0.4589; intra-class correlation coefficient: 0.14.

Table S10. Determinants of urinary DEHP metabolites (µg/L): multiple regression model in children.

**Parameters**	**Estimate (95%CI) for change (multiplicative factor)**	***p*-value**	**Overall *p*-value**
**Urinary creatinine level**			
300-900 mg/L	0.46 (0.42,00.51)	<0.001	<0.001
900,01500 mg/L	0.75 (0.69, 0.83)	<0.001	
1500, 3000 mg/L	1.00		
**Gender**			
Boys	1.02 (0.95, 1.09)	0.63	0.63
Girls	1.00		
**Age**			
5-8 years	1.19 (1.11, 1.27)	<0.001	<0.001
9-11 years	1.00		
**Ice cream consumption**			
Several times/week	1.12 (1.01, 1.25)	0.04	0.04
Once/week or less	1.00		
**Gum consumption**			
Several times/week	1.10 (1.02, 1.18)	0.01	0.01
Once/week or less	1.00		

Urinary DEHP metabolites: sum of MEHP, 5OH-MEHP and 5oxo-MEHP.

Number of observations in model: N=1621. Cluster variance: 0.15 (p=0.004); residual variance: 0.4577; intra-class correlation coefficient: 0.24.

Table S11. Determinants of urinary DEHP metabolites (µg/L): multiple regression model in mothers.

Parameters	Estimate (95%CI) for change (multiplicative factor)	*p*-value	Overall *p*-value
**Urinary creatinine level**			
300-900 mg/L	0.35 (0.32, 0.38)	<0.001	<0.001
900-1500 mg/L	0.62 (0.57, 0.68)	<0.001	
1500-3000 mg/L	1.00		
**Age**			
≤35 years	0.96 (0.87, 1.06)	0.38	0.22
35-40 years	0.99 (0.91, 1.07)	0.79	
>40 years	1.00		
**Urine sampling period**			
<7 hours	0.87 (0.79, 0.97)	0.01	0.02
7-9 hours	0.97 (0.88, 1.06)	0.48	
≥ 9 hours	1.00		
**Use of personal care products**			
High use	0.91(0.84, 0.98)	0.01	0.01
Moderate to low use	1.00		
**Educational level of the family**			
Primary	1.20 (1.05, 1.37)	0.006	0.02
Secondary	1.04 (0.96, 1.13)	0.31	
Tertiary	1.00		

Urinary DEHP metabolites: sum of MEHP, 5OH-MEHP and 5oxo-MEHP.

Number of observations in model: N=1723. Cluster variance: 0.12 (p=0.004); residual variance: 0.5263; intra-class correlation coefficient: 0.18.

Table S12. Determinants of urinary MnBP (µg/L): multiple regression model in children.

Parameters	Estimate (95%CI) for change (multiplicative factor)	*p*-value	Overall *p*-value
**Urinary creatinine level**			
300-900 mg/L	0.45 (0.41, 0.50)	<0.001	<0.001
900-1500 mg/L	0.73 (0.66, 0.81)	<0.001	
1500-3000 mg/L	1.00		
**Gender**			
Boys	0.91 (0.85, 0.98)	0.008	0.008
Girls	1.00		
**Age**			
5-8 years	1.15 (1.07, 1.24)	<0.001	<0.001
9-11 years	1.00		
**PVC in floors/walls**			
Yes	1.19 (1.08, 1.32)	<0.001	<0.001
No	1.00		
**Educational level of the family**			
Primary	0.91 (0.81, 1.03)	0.16	0.02
Secondary	0.89 (0.82, 0.97)	0.006	
Tertiary	1.00		

Number of observations in model: N=1330. Cluster variance: 0.16 (p=0.009); residual variance: 0.4396; intra-class correlation coefficient: 0.27.

Table S13. Determinants of urinary MnBP (µg/L): multiple regression model in mothers.

Parameters	Estimate (95%CI) for change (multiplicative factor)	*p*-value	Overall *p*-value
**Urinary creatinine level**			
300-900 mg/L	0.35 (0.32, 0.38)	<0.001	<0.001
900-1500 mg/L	0.60 (0.55, 0.66)	<0.001	
1500-3000 mg/L	1.00		
**Age**			
≤35 years	0.81 (0.73, 0.89)	<0.001	<0.001
35-40 years	0.93 (0.86, 1.01)	0.10	
>40 years	1.00		
**BMI**			
Normal weight	1.15 (1.02, 1.29)	0.02	0.047
Overweight	1.09 (0.96, 1.24)	0.17	
Obese	1.00		
**Ice cream consumption**			
Several times/month	1.10 (1.01, 1.19)	0.02	0.02 *
Once/month or less	1.00		
**Use of personal care products**			
High	0.92 (0.86, 0.99)	0.03	0.03
Moderate/low	1.00		

Number of observations in model: N=1304. Cluster variance: 0.12 (p=0.01); residual variance: 0.415; intra-class correlation coefficient: 0.22.

- not significant (p=0.22) after weighing for unequal numbers per country.

Table S14. Determinants of urinary MBzP (µg/L): multiple regression model in children.

Parameters	Estimate (95%CI) for change (multiplicative factor)	*p*-value	Overall *p*-value
**Urinary creatinine level**			
300-900 mg/L	0.41 (0.37, 0.47)	<0.001	<0.001
900-1500 mg/L	0.69 (0.62, 0.78)	<0.001	
1500-3000 mg/L	1.00		
**Gender**			
boys	1.05 (0.96, 1.14)	0.30	0.30
girls	1.00		
**Age**			
5-8 years	1.15 (1.06, 1.26)	0.001	0.001
9-11 years	1.00		
**Morning urine**			
Yes	1.98 (1.17, 3.36)	0.01	0.01
No	1.00		
**Ice cream consumption**			
Several times/week	1.18 (1.02, 1.36)	0.03	0.03
Once/week or less	1.00		
**PVC in floors/walls**			
Yes	1.50 (1.34, 1.68)	<0.001	<0.001
No	1.00		

Number of observations in model: N=1722. Cluster variance: 0.17 (p=0.004); residual variance: 0.818; intra-class correlation coefficient: 0.17.

Table S15. Determinants of urinary MBzP metabolites (µg/L): multiple regression model in mothers.

**Parameters**	**Estimate (95%CI) for change (multiplicative factor)**	***p*-value**	**Overall *p*-value**
**Urinary creatinine level**			
300-900 mg/L	0.33 (0.30, 0.37)	<0.001	<0.001
900-1500 mg/L	0.59 (0.54, 0.65)	<0.001	
1500-3000 mg/L	1.00		
**Age**			
≤35 years	1.01 (0.91, 1.13)	0.82	0.20
35-40 years	1.08 (0.99, 1.19)	0.10	
>40 years	1.00		
**Ice cream consumption**			
Several times/month	1.13 (1.03, 1.24)	0.01	0.01
Once/month or less	1.00		
**PVC in floors/walls**			
Yes	1.32 (1.19, 1.47)	<0.001	<0.001
No	1.00		

Number of observations in model: N=1718. Cluster variance: 0.19 (p=0.004); residual variance: 0.7003; intra-class correlation coefficient: 0.21.

Table S16. Determinants of urinary MEP (µg/L): multiple regression model in children.

**Parameters**	**Estimate (95%CI) for change (multiplicative factor)**	***p*-value**	**Overall *p*-value**
**Urinary creatinine level**			
300-900 mg/L	0.41 (0.36, 0.47)	<0.001	<0.001
900-1500 mg/L	0.68 (0.61, 0.77)	<0.001	
1500-3000 mg/L	1.00		
**Gender**			
Boys	0.96 (0.87, 1.05)	0.36	0.36
Girls	1.00		
**Age**			
5-8 years	1.15 (1.04, 1.26)	0.004	0.004
9-11 years	1.00		
**Urine sampling period**			
<10 hours	1.20 (1.06, 1.35)	0.003	0.01
10-11 hours	1.14 (1.02, 1.29)	0.02	
≥ 11 hours	1.00		
**Use of personal care products**			
Moderate to high use	1.24 (1.13, 1.37)	<0.001	<0.001
Low use	1.00		
**Educational level of the family**			
Primary	0.91 (0.81, 1.03)	0.16	0.02
Secondary	0.89 (0.82, 0.97)	0.006	
Tertiary	1.00		

Number of observations in model: N=1601. Cluster variance: 0.16 (p=0.006); residual variance: 1.2581; intra-class correlation coefficient: 0.11.

Table S17. Determinants of urinary MEP (µg/L): multiple regression model in mothers.

**Parameters**	**Estimate (95%CI) for change (multiplicative factor)**	***p*-value**	**Overall *p*-value**
**Urinary creatinine level**			
300-900 mg/L	0.32 (0.28, 0.37)	<0.001	<0.001
900-1500 mg/L	0.63 (0.55, 0.72)	<0.001	
1500-3000 mg/L	1.00		
**Age**			
≤35 years	0.87 (0.75, 1.02)	0.09	0.08
35-40 years	0.87 (0.76, 0.99)	0.04	
>40 years	1.00		
**Gum consumption**			
Several times/week	1.19 (1.06, 1.34)	0.003	0.003
Once/week or less	1.00		
**Use of personal care products**			
High use	1.40 (1.25, 1.56)	<0.001	<0.001
Moderate to low use	1.00		

Number of observations in model: N=1601. Cluster variance: 0.16 (p=0.006); residual variance: 1.2581; intra-class correlation coefficient: 0.11.

Table S18. Determinants of urinary MiBP (μg/L): multiple regression model in children.

**Parameters**	**Estimate (95%CI) for change (multiplicative factor)**	***p*-value**	**Overall *p*-value**
**Urinary creatinine level**			
300-900 mg/L	0.45 (0.40, 0.50)	<0.001	<0.001
900-1500 mg/L	0.72 (0.65, 0.80)	<0.001	
1500-3000 mg/L	1.00		
**Gender**			
boys	0.92 (0.85, 0.99)	0.03	0.03
girls	1.00		
**Age**			
5-8 years	1.19 (1.10, 1.28)	<0.001	<0.001
9-11 years	1.00		
**Use of personal care products**			
Moderate to high use	1.13 (1.03, 1.23)	0.007	0.007
Low use	1.00		
**PVC in floors/walls**			
Yes	1.22 (1.09, 1.35)	<0.001	<0.001
No	1.00		

Number of observations in model: N=1314. Cluster variance: 0.16 (p=0.009); residual variance: 0.4985; intra-class correlation coefficient: 0.24.

Table S19. Determinants of urinary MiBP (µg/L): multiple regression model in mothers.

**Parameters**	**Estimate (95%CI) for change (multiplicative factor)**	***p*-value**	**Overall *p*-value**
**Urinary creatinine level**			
300-900 mg/L	0.38 (0.35, 0.41)	<0.001	<0.001
900-1500 mg/L	0.61 (0.56, 0.67)	<0.001	
1500-3000 mg/L	1.00		
**Age**			
≤35 years	0.98 (0.88, 1.08)	0.65	0.70
35-40 years	1.02 (0.94, 1.10)	0.69	
>40 years	1.00		
**Renovation in house**			
Yes	1.08 (1.00, 1.16)	0.04	0.04*
No	1.00		
**PVC in floors/walls**			
Yes	1.15 (1.04, 1.26)	0.006	0.006
No	1.00		
**Educational level of the family**			
Primary	1.09 (0.97, 1.23)	0.16	0.04
Secondary	1.11 (1.02, 1.21)	0.01	
Tertiary	1.00		

Number of observations in model: N=1325. Cluster variance: 0.14 (p=0.010); residual variance: 0.4162; intra-class correlation coefficient: 0.25.

*Not significant (p=0.11) after weighing for unequal numbers per country.

3. Comparison of results between countries

The results of the comparison between countries are given in Supplemental Material, Table S20 to Table S35.

Table S20. Comparison of mean mercury levels in hair (µg/g) between 17 European countries, results in children, unadjusted and adjusted data.

Country	N	LOQ	% <LOQ	GM (95% CI), unadjusted	*p*-value[a]	GM (95% CI), adj. for age and gender	*p*-value[a]
ALL	1836	<0.001-0.137	14.1%	0.145 (0.139, 0.151)	<0.001	0.144 (0.139, 0.151)	<0.001
Belgium	127	0.08	19.7%	0.204 (0.713, 0.242)	<0.001	0.204 (0.173, 0.241)	<0.001
Switzerland	120	0.02	21.7%	0.077 (0.065, 0.091)	<0.001	0.076 (0.065, 0.090)	<0.001
Cyprus	60	0.01	1.7%	0.326 (0.257, 0.413)	<0.001	0.326 (0.257, 0.413)	<0.001
Czech Republic	120	0.014	0.0%	0.098 (0.083, 0.116)	<0.001	0.098 (0.083, 0.116)	<0.001
Germany	120	0.003	0.0%	0.055 (0.047, 0.065)	<0.001	0.055 (0.046, 0.065)	<0.001
Denmark	144	0.04	2.1%	0.249 (0.211, 0.295)	<0.001	0.250 (0.211, 0.295)	<0.001
Spain	120	0.01	0.0%	0.884 (0.747, 1.046)	<0.001	0.884 (0.747, 1.046)	<0.001
Hungary	119	0.015	48.7%	0.025 (0.021, 0.029)	<0.001	0.025 (0.021, 0.029)	<0.001
Ireland	120	0.07	37.0%	0.097 (0.082, 0.114)	<0.001	0.097 (0.082, 0.114)	<0.001
Luxembourg	56	0.005	0.0%	0.180 (0.142, 0.228)	0.06	0.181 (0.142, 0.229)	0.06
Poland	120	0.01	1.7%	0.070 (0.060, 0.083)	<0.001	0.070 (0.060, 0.083)	<0.001
Portugal	120	0.08	0.0%	1.035 (0.875, 1.225)	<0.001	1.033 (0.873, 1.222)	<0.001
Romania	120	0.137	8.7%	0.085 (0.072, 0.101)	<0.001	0.085 (0.072, 0.101)	<0.001
Sweden	100	<0.001	0.0%	0.181 (0.153, 0.214)	0.007	0.181 (0.153, 0.214)	0.007
Slovenia	120	0.01	0.0%	0.168 (0.142, 0.199)	0.07	0.169 (0.142, 0.200)	0.06
Slovak Republic	129	0.016	0.0%	0.092 (0.078, 0.109)	<0.001	0.092 (0.078, 0.109)	<0.001
United Kingdom	21	0.01	0.0%	0.193 (0.163, 0.228)	<0.001	0.192 (0.163, 0.228)	<0.001

LOQ: limit of quantification; GM: geometric mean; CI: confidence interval.

[a]*p*-values for comparison between countries are calculated as follow: in a first step, overall significance of country is tested by a linear regression model (see *p*-value ALL in first row); in case of an overall significant difference between the countries ($p<0.05$), a *post hoc*

analysis is done and the mean exposure level in each country is compared with the European exposure value (see *p*-value per country in the following rows).

Table S21. Comparison of mean mercury levels (µg/g) in hair between 17 European countries, results in mothers, unadjusted and adjusted data.

Country	N	LOQ	% <LOQ	GM (95% CI), unadjusted	*p*-value	GM (95% CI), adjusted for age	*p*-value
ALL	1836	<0.001-0.137	14.1%	0.225 (0.216, 0.234)	<0.001	0.220 (0.212, 0.229)	<0.001
Belgium	127	0.08	19.7%	0.383 (0.327, 0.450)	<0.001	0.368 (0.313, 0.431)	<0.001
Switzerland	120	0.02	21.7%	0.163 (0.139, 0.191)	<0.001	0.153 (0.131, 0.180)	<0.001
Cyprus	60	0.01	1.7%	0.463 (0.369, 0.580)	<0.001	0.462 (0.369, 0.578)	<0.001
Czech Republic	120	0.014	0.0%	0.155 (0.132, 0.182)	<0.001	0.156 (0.133, 0.183)	<0.001
Germany	120	0.003	0.0%	0.113 (0.096, 0.133)	<0.001	0.107 (0.092, 0.126)	<0.001
Denmark	144	0.04	2.1%	0.420 (0.358, 0.493)	<0.001	0.391 (0.333, 0.458)	<0.001
Spain	120	0.01	0.0%	1.592 (1.357, 1.869)	<0.001	1.468 (1.267, 1.744)	<0.001
Hungary	119	0.015	48.7%	0.038 (0.032, 0.044)	<0.001	0.039 (0.033, 0.045)	<0.001
Ireland	120	0.07	37.5%	0.165 (0.141, 0.194)	<0.001	0.162 (0.139, 0.190)	<0.001
Luxembourg	56	0.005	0.0%	0.416 (0.332, 0.522)	<0.001	0.387 (0.308, 0.485)	<0.001
Poland	120	0.01	1.7%	0.131 (0.111, 0.153)	<0.001	0.135 (0.116, 0.159)	<0.001
Portugal	120	0.08	0.0%	1.203 (1.025, 1.412)	<0.001	1.200 (1.023, 1.406)	<0.001
Romania	120	0.137	81.7%	0.095 (0.081, 0.111)	<0.001	0.100 (0.085, 0.117)	<0.001
Sweden	100	<0.001	0.0%	0.260 (0.222, 0.305)	0.07	0.252 (0.215, 0.295)	0.09
Slovenia	120	0.01	0.0%	0.263 (0.224, 0.309)	0.046	0.255 (0.217, 0.299)	0.06
Slovak Republic	129	0.016	0.0%	0.129 (0.110, 0.152)	<0.001	0.132 (0.112, 0.154)	<0.001
United Kingdom	21	0.01	0.0%	0.163 (0.139, 0.192)	<0.001	0.153 (0.130, 0.180)	<0.001

LOQ: limit of quantification; GM: geometric mean; CI: confidence interval.

Table S22. Comparison of mean urinary cotinine levels between 17 European countries, results in children, unadjusted and adjusted data.

Country	N	LOQ (µg/L)	% <LOQ	Urinary cotinine (µg/L) GM (95% CI) unadjusted	*p*-value	Urinary cotinine (µg/L) GM (95% CI), adjusted[a]	*p*-value	Urinary cotinine (µg/g creatinine) GM (95% CI), unadjusted	*p*-value	Urinary cotinine (µg/g creatinine) GM (95% CI), adjusted[b]	*p*-value
ALL	1818	0.1–1.2	57.6%	0.797 (0.759, 0.837)	<0.001	0.800 (0.760, 0.843)	<0.001	0.774 (0.736, 0.815)	<0.001	0.774 (0.735, 0.815)	<0.001
Belgium	125	0.7	65.6%	0.634 (0.521, 0.771)	0.02	0.629 (0.517, 0.766)	0.01	0.566 (0.461, 0.696)	0.002	0.566 (0.461, 0.695)	0.002
Switzerland	119	1	98.3%	0.506 (0.416, 0.615)	<0.001	0.508 (0.418, 0.619)	<0.001	0.487 (0.396, 0.598)	<0.001	0.484 (0.394, 0.594)	<0.001
Cyprus	60	0.8	53.3%	0.841 (0.638, 1.110)	0.70	0.842 (0.638, 1.111)	0.72	0.804 (0.601, 1.076)	0.80	0.804 (0.602, 1.075)	0.79
Czech Republic	120	0.3	0.0%	1.585 (1.303, 1.928)	<0.001	1.602 (1.361, 1.950)	<0.001	1.615 (1.315, 1.984)	<0.001	1.612 (1.313, 1.979)	<0.001
Germany	120	0.1	13.3%	0.308 (0.253, 0.374)	<0.001	0.305 (0.251, 0.371)	<0.001	0.280 (0.228, 0.344)	<0.001	0.277 (0.225, 0.340)	<0.001
Denmark	144	0.75	64.6%	0.649 (0.534, 0.790)	0.03	0.658 (0.541, 0.801)	0.04	0.679 (0.553, 0.835)	0.20	0.682 (0.555, 0.837)	0.21
Spain	119	0.1	0.0%	1.459 (1.200, 1.775)	<0.001	1.485 (1.219, 1.810)	<0.001	1.596 (1.299, 1.961)	<0.001	1.595 (1.299, 1.958)	<0.001
Hungary	117	0.3	0.0%	1.800 (1.480, 2.190)	<0.001	1.7736 (1.460, 2.161)	<0.001	1.593 (1.297, 1.957)	<0.001	1.592 (1.297, 1.954)	<0.001
Ireland	120	0.48	61.7%	0.712 (0.585, 0.866)	0.24	0.708 (0.582, 0.862)	0.21	0.659 (0.537, 0.810)	0.11	0.659 (0.537, 0.809)	0.11
Luxembourg	59	0.59	83.1%	0.391 (0.297, 0.516)	<0.001	0.397 (0.301, 0.524)	<0.001	0.390 (0.292, 0.522)	<0.001	0.391 (0.292, 0.522)	<0.001
Poland	115	0.8	34.8%	1.558 (1.281, 1.895)	<0.001	1.568 (1.288, 1.909)	<0.001	1.596 (1.299, 1.961)	<0.001	1.598 (1.301, 1.961)	<0.001
Portugal	116	0.7	39.7%	1.073 (0.882, 1.305)	0.002	1.093 (0.897, 1.333)	<0.001	1.194 (0.972, 1.466)	<0.001	1.191 (0.970, 1.463)	<0.001
Romania	119	0.7	26.1%	1.953 (1.605, 2.375)	<0.001	1.942 (1.597, 2.363)	<0.001	1.723 (1.402, 2.116)	<0.001	1.723 (1.404, 2.116)	<0.001
Sweden	97	0.3	84.5%	0.199 (0.163, 0.242)	<0.001	0.202 (0.165, 0.246)	<0.001	0.224 (0.183, 0.276)	<0.001	0.224 (0.182, 0.275)	<0.001
Slovenia	120	0.7	74.2%	0.532 (0.438, 0.648)	<0.001	0.529 (0.434, 0.644)	<0.001	0.442 (0.360, 0.543)	<0.001	0.446 (0.363, 0.549)	<0.001
Slovak Republic	127	0.3	0.0%	1.104 (0.907, 1.342)	<0.001	1.085 (0.892, 1.320)	0.002	0.911 (0.742, 1.119)	0.11	0.908 (0.740, 1.115)	0.11
United Kingdom	21	1.2	95.2%	0.641 (0.527, 0.780)	0.02	0.661 (0.542, 0.806)	0.049	0.712 (0.580, 0.875)	0.41	0.712 (0.580, 0.874)	0.41

LOQ: limit of quantification; GM: geometric mean; CI: confidence interval.

[a]Adjusted for age, gender and creatinine. [b]Adjusted for age and gender.

Table S23. Comparison of mean urinary cotinine levels between 17 European countries, results in mothers, unadjusted and adjusted data.

Country	N	LOQ (µg/L)	% <LOQ	Urinary cotinine (µg/L) GM (95% CI), unadjusted	*p*-value	Urinary cotinine (µg/L) GM (95% CI), adjusted[a]	*p*-value	Urinary cotinine (µg/g creatinine) GM (95% CI), unadjusted	*p*-value	Urinary cotinine (µg/g creatinine) GM (95% CI), adjusted[b]	*p*-value
ALL	1800	0.1-1.2	62.4%	2.75 (2.41, 3.14)	<0.001	2.85 (2.49, 3.27)	<0.001	2.45 (2.14, 2.80)	<0.001	2.53 (2.21, 2.90)	<0.001
Belgium	125	0.7	69.6%	1.15 (0.67, 1.97)	<0.001	1.26 (0.74, 2.15)	0.002	1.02 (0.60, 1.76)	<0.001	1.10 (0.64, 1.88)	0.002
Switzerland	117	1	94.0%	0.71 (0.42, 1.21)	<0.001	0.84 (0.49, 1.45)	<0.001	0.73 (0.43, 1.26)	<0.001	0.82 (0.48, 1.40)	<0.001
Cyprus	59	0.8	40.7%	2.87 (1.35, 6.12)	0.91	2.82 (1.33, 6.00)	0.98	2.47 (1.16, 5.27)	0.98	2.49 (1.17, 5.29)	0.96
Czech Republic	117	0.3	0.0%	3.80 (2.22, 6.50)	0.22	3.77 (2.21, 6.44)	0.29	3.58 (2.09, 6.11)	0.15	3.51 (2.06, 6.00)	0.22
Germany	116	0.1	13.8%	0.92 (0.54, 1.57)	<0.001	1.00 (0.59, 1.72)	<0.001	0.81 (0.48, 1.39)	<0.001	0.88 (0.52, 1.51)	<0.001
Denmark	143	0.75	51.7%	1.54 (0.90, 2.63)	0.03	1.87 (1.09, 3.21)	0.11	1.57 (0.92, 2.68)	0.09	1.78 (1.04, 3.06)	0.19
Spain	118	0.1	1.7%	8.32 (4.87, 14.2)	<0.001	9.59 (5.60, 16.4)	<0.001	7.79 (4.56, 13.3)	<0.001	8.78 (5.12, 15.1)	<0.001
Hungary	115	0.3	0.0%	7.97 (4.67, 13.6)	<0.001	7.19 (1.09, 3.21)	<0.001	6.40 (3.74, 10.9)	<0.001	6.05 (3.54, 10.3)	<0.001
Ireland	120	0.48	52.5%	3.84 (2.24, 6.56)	0.21	3.86 (2.27, 6.58)	0.25	3.28 (1.92, 5.60)	0.27	3.37 (1.98, 5.75)	0.28
Luxembourg	56	0.59	83.9%	0.47 (0.22, 1.00)	<0.001	0.56 (0.26, 1.19)	<0.001	0.50 (0.24, 1.08)	<0.001	0.56 (0.26, 1.20)	<0.001
Poland	119	0.8	36.1%	6.67 (3.91, 11.4)	<0.001	6.22 (3.64, 10.6)	0.003	6.07 (3.56, 10.4)	<0.001	5.71 (3.34, 9.75)	0.002
Portugal	117	0.7	35.0%	11.4 (6.67, 19.5)	<0.001	10.9 (6.40, 18.6)	<0.001	9.15 (5.36, 15.6)	<0.001	9.09 (5.33, 15.5)	<0.001
Romania	117	0.7	18.8%	17.5 (10.3, 30.0)	<0.001	14.9 (8.70, 25.6)	<0.001	13.6 (7.99, 23.3)	<0.001	12.3 (7.2, 21.1)	<0.001
Sweden	96	0.3	59.4%	1.67 (0.98, 2.85)	0.06	1.80 (1.06, 3.08)	0.08	1.57 (0.92, 2.69)	0.09	1.64 (0.96, 2.81)	0.10
Slovenia	119	0.7	59.7%	1.75 (1.02, 2.98)	0.09	1.79 (1.05, 3.06)	0.08	1.36 (0.79, 2.31)	0.03	1.44 (0.84, 2.46)	0.03
Slovak Republic	125	0.3	0.0%	3.00 (1.75, 5.12)	0.75	2.82 (1.65, 4.81)	0.96	2.43 (1.42, 4.16)	0.98	2.37 (1.38, 4.03)	0.79
United Kingdom	21	1.2	90.5%	0.71 (0.41, 1.21)	<0.001	0.84 (0.49, 1.45)	<0.001	0.68 (0.40, 1.16)	<0.001	0.77 (0.45, 1.33)	<0.001

LOQ: limit of quantification; GM: geometric mean; CI: confidence interval.

[a]Adjusted for age and creatinine. [b]Adjusted for age.

Table S24. Comparison of mean urinary cadmium levels between 17 European countries, results in children, unadjusted and adjusted data.

Country	N	LOQ (µg/L)	% <LOQ	Urinary cadmium (µg/L) GM (95% CI), unadjusted	*p*-value	Urinary cadmium (µg/L) GM (95% CI), adjusted[a]	*p*-value	Urinary cadmium (µg/g creatinine) GM (95% CI), unadjusted	*p*-value	Urinary cadmium (µg/g creatinine) GM (95% CI), adjusted[b]	*p*-value
ALL	1698	<0.001-0.2	29.9%	0.071 (0.069, 0.074)	<0.001	0.076 (0.073, 0.078)	<0.001	0.070 (0.067, 0.072)	<0.001	0.070 (0.067, 0.072)	<0.001
Belgium	125	0.01	13.6%	0.044 (0.039, 0.050)	<0.001	0.046 (0.041, 0.052)	<0.001	0.039 (0.035, 0.045)	<0.001	0.039 (0.035, 0.045)	<0.001
Switzerland	119	0.07	31.9%	0.076 (0.067, 0.087)	0.31	0.080 (0.071, 0.091)	0.32	0.073 (0.065, 0.083)	0.41	0.073 (0.064, 0.083)	0.43
Cyprus	60	0.2	91.7%	0.108 (0.090, 0.130)	<0.001	0.115 (0.097, 0.137)	<0.001	0.103 (0.086, 0.123)	<0.001	0.103 (0.086, 0.123)	<0.001
Czech Republic	120	0.05	8.3%	0.109 (0.096, 0.124)	<0.001	0.118 (0.104, 0.133)	<0.001	0.111 (0.098, 0.126)	<0.001	0.111 (0.098, 0.126)	<0.001
Denmark	142	0.025	66.9%	0.021 (0.019, 0.024)	<0.001	0.023 (0.021, 0.026)	<0.001	0.022 (0.020, 0.025)	<0.001	0.022 (0.020, 0.025)	<0.001
Spain	119	0.025	26.9%	0.042 (0.037, 0.048)	<0.001	0.047 (0.042, 0.053)	<0.001	0.046 (0.040, 0.052)	<0.001	0.046 (0.040, 0.052)	<0.001
Hungary	117	0.1	29.9%	0.132 (0.116, 0.150)	<0.001	0.130 (0.115, 0.147)	<0.001	0.117 (0.103, 0.132)	<0.001	0.117 (0.103, 0.132)	<0.001
Ireland	120	0.064	44.2%	0.066 (0.058, 0.075)	0.19	0.068 (0.060, 0.076)	0.06	0.061 (0.054, 0.069)	0.03	0.061 (0.054, 0.069)	0.03
Luxembourg	59	0.05	0.0%	0.144 (0.119, 0.173)	<0.001	0.154 (0.130, 0.184)	<0.001	0.143 (0.120, 0.171)	<0.001	0.143 (0.120, 0.171)	<0.001
Poland	115	0.012	0.0%	0.126 (0.111, 0.144)	<0.001	0.135 (0.119, 0.153)	<0.001	0.129 (0.114, 0.147)	<0.001	0.129 (0.114, 0.147)	<0.001
Portugal	116	0.01	12.1%	0.039 (0.034, 0.045)	<0.001	0.045 (0.040, 0.051)	<0.001	0.044 (0.039, 0.050)	<0.001	0.044 (0.039, 0.050)	<0.001
Romania	119	0.051	95.0%	0.027 (0.024, 0.031)	<0.001	0.026 (0.023, 0.030)	<0.001	0.024 (0.021, 0.027)	<0.001	0.024 (0.021, 0.027)	<0.001
Sweden	99	<0.001	0.0%	0.078 (0.068, 0.089)	0.17	0.090 (0.079, 0.102)	0.005	0.088 (0.077, 0.099)	<0.001	0.088 (0.077, 0.099)	<0.001
Slovenia	120	0.06	35.8%	0.081 (0.071, 0.092)	0.05	0.077 (0.068, 0.087)	0.77	0.067 (0.059, 0.076)	0.57	0.067 (0.059, 0.076)	0.53
Slovak Republic	127	0.05	2.4%	0.149 (0.131, 0.170)	<0.001	0.145 (0.128, 0.164)	<0.001	0.123 (0.108, 0.139)	<0.001	0.123 (0.109, 0.139)	<0.001
United Kingdom	21	0.01	0.0%	0.144 (0.126, 0.164)	<0.001	0.166 (0.147, 0.188)	<0.001	0.160 (0.141, 0.181)	<0.001	0.159 (0.140, 0.180)	<0.001

LOQ: limit of quantification; GM: geometric mean; CI: confidence interval.

[a]Adjusted for age, gender and creatinine. [b]Adjusted for age and gender.

Table S25. Comparison of mean urinary cadmium levels between 17 European countries, results in mothers, unadjusted and adjusted data.

Country	N	LOQ (µg/L)	% <LOQ	Urinary cadmium (µg/L) GM (95% CI), unadjusted	*p*-value	Urinary cadmium (µg/L) GM (95% CI), adjusted[a]	*p*-value	Urinary cadmium (µg/g creatinine) GM (95% CI), unadjusted	*p*-value	Urinary cadmium (µg/g creatinine) GM (95% CI), adjusted[b]	*p*-value
ALL	1685	<0.001-0.2	6.2%	0.219 (0.211, 0.228)	<0.001	0.230 (0.220, 0.241)	<0.001	0.196 (0.189, 0.202)	<0.001	0.204 (0.195, 0.213)	<0.001
Belgium	125	0.01	0.8%	0.205 (0.177, 0.238)	0.35	0.224 (0.197, 0.255)	0.66	0.183 (0.162, 0.207)	0.27	0.194 (0.171, 0.219)	0.38
Switzerland	117	0.07	6.0%	0.189 (0.163, 0.219)	0.04	0.224 (0.197, 0.255)	0.66	0.196 (0.173, 0.221)	0.99	0.205 (0.181, 0.232)	0.94
Cyprus	59	0.2	52.5%	0.182 (0.148, 0.224)	0.08	0.183 (0.153, 0.219)	0.009	0.157 (0.132, 0.187)	0.01	0.165 (0.139, 0.197)	0.01
Czech Republic	117	0.05	0.9%	0.227 (0.196, 0.263)	0.65	0.259 (0.228, 0.295)	0.05	0.213 (0.189, 0.242)	0.15	0.235 (0.208, 0.266)	0.02
Denmark	142	0.025	8.5%	0.115 (0.099, 0.133)	<0.001	0.132 (0.116, 0.150)	<0.001	0.118 (0.104, 0.134)	<0.001	0.119 (0.105, 0.135)	<0.001
Spain	118	0.025	1.7%	0.230 (0.198, 0.266)	0.53	0.212 (0.187, 0.241)	0.18	0.215 (0.190, 0.243)	0.12	0.196 (0.173, 0.220)	0.50
Hungary	115	0.1	19.1%	0.188 (0.162, 0.217)	0.03	0.183 (0.161, 0.207)	<0.001	0.150 (0.133, 0.170)	<0.001	0.159 (0.141, 0.180)	<0.001
Ireland	120	0.064	4.2%	0.314 (0.271, 0.363)	<0.001	0.296 (0.261, 0.207)	<0.001	0.268 (0.237, 0.303)	<0.001	0.265 (0.235, 0.300)	<0.001
Luxembourg	56	0.05	0.0%	0.200 (0.162, 0.246)	0.37	0.249 (0.208, 0.298)	0.38	0.215 (0.180, 0.256)	0.29	0.231 (0.194, 0.275)	0.15
Poland	119	0.012	0.0%	0.416 (0.359, 0.482)	<0.001	0.453 (0.399, 0.514)	<0.001	0.379 (0.334, 0.429)	<0.001	0.408 (0.361, 0.461)	<0.001
Portugal	117	0.01	0.9%	0.201 (0.174, 0.233)	0.23	0.186 (0.164, 0.211)	<0.001	0.161 (0.143, 0.183)	0.002	0.160 (0.142, 0.181)	<0.001
Romania	117	0.051	16.2%	0.192 (0.165, 0.222)	0.06	0.187 (0.164, 0.121)	<0.001	0.149 (0.132, 0.169)	<0.001	0.160 (0.142, 0.181)	<0.001
Sweden	98	<0.001	0.0%	0.147 (0.127, 0.170)	<0.001	0.175 (0.154, 0.199)	<0.001	0.139 (0.123, 0.157)	<0.001	0.152 (0.134, 0.173)	<0.001
Slovenia	119	0.06	2.5%	0.298 (0.257, 0.345)	<0.001	0.289 (0.255, 0.329)	<0.001	0.231 (0.204, 0.262)	0.006	0.244 (0.216, 0.276)	0.002
Slovak Republic	125	0.05	0.0%	0.294 (0.254, 0.341)	<0.001	0.306 (0.269, 0.348)	<0.001	0.239 (0.211, 0.271)	<0.001	0.261 (0.231, 0.296)	<0.001
United Kingdom	21	0.01	0.0%	0.244 (0.211, 0.283)	0.14	0.267 (0.234, 0.304)	0.02	0.234 (0.207, 0.265)	0.003	0.241 (0.212, 0.274)	0.006

LOQ: limit of quantification; GM: geometric mean; CI: confidence interval.

[a]Adjusted for age, smoking and creatinine. [b]Adjusted for age and smoking.

Table S26. Comparison of mean urinary level of DEHP metabolites between 17 European countries, results in children, unadjusted and adjusted data.

Country	N	LOQ MEHP (µg/L)	LOQ 5OH-MEHP (µg/L)	LOQ 5oxo-MEHP (µg/L)	% <LOQ	Urinary DEHP (µg/L) GM (95% CI), unadjusted	*p*-value	Urinary DEHP (µg/L) GM (95% CI), adjusted[a]	*p*-value	Urinary DEHP (µg/g creatinine) GM (95% CI), unadjusted	*p*-value	Urinary DEHP (µg/g creatinine) GM (95% CI), adjusted[b]	*p*-value
ALL	1816	0.3-3.9	0.1-9.2	0.1-6.2	14.4%	47.6 (46.0, 49.3)	<0.001	50.0 (48.4, 51.7)	<0.001	46.2 (44.9, 47.7)	<0.001	46.2 (44.8, 47.6)	<0.001
Belgium	125	0.5	0.1	0.1	4.8%	36.7 (32.1, 42.1)	<0.001	37.3 (32.9, 42.2)	<0.001	32.8 (29.0, 37.1)	<0.001	32.8 (29.1, 37.0)	<0.001
Switzerland	119	3.9	9.2	6.2	85.7%	26.9 (23.4, 30.8)	<0.001	28.1 (24.8, 31.9)	<0.001	25.8 (22.9, 29.2)	<0.001	25.1 (22.8, 29.0)	<0.001
Cyprus	60	2.6	2.5	1.6	46.7%	23.8 (19.6, 28.8)	<0.001	25.0 (21.0, 29.8)	<0.001	22.7 (19.1, 27.0)	<0.001	22.7 (19.2, 26.9)	<0.001
Czech Republic	120	2	0.61	0.24	27.5%	65.7 (57.3, 75.2)	<0.001	71.1 (62.7, 80.5)	<0.001	67.0 (59.3, 75.7)	<0.001	66.8 (59.2, 75.4)	<0.001
Germany	120	0.5	0.2	0.2	6.7%	39.2 (34.2, 44.9)	0.004	39.5 (34.9, 44.8)	<0.001	35.6 (31.5, 40.2)	<0.001	35.1 (31.1, 39.6)	<0.001
Denmark	142	0.14	0.91	0.67	4.2%	37.3 (32.6, 42.8)	<0.001	40.9 (36.1, 46.4)	<0.001	38.8 (34.3, 43.9)	0.004	39.1 (34.6, 44.1)	0.005
Spain	119	0.5	0.2	0.2	0.0%	64.6 (56.4, 74.0)	<0.001	73.4 (64.7, 83.2)	<0.001	70.7 (62.6, 79.9)	<0.001	70.6 (62.6, 79.7)	<0.001
Hungary	117	2	0.61	0.24	24.8%	60.8 (53.1, 69.7)	<0.001	58.7 (51.8, 66.5)	0.009	53.8 (47.6, 60.8)	0.012	53.7 (47.6, 60.6)	0.011
Ireland	120	1	0.5	0.5	0.0%	58.8 (51.4, 67.4)	0.002	59.6 (52.6, 67.5)	0.004	54.5 (48.2, 61.6)	0.006	54.4 (48.2, 61.4)	0.006
Luxembourg	59	0.5	0.1	0.1	6.8%	23.8 (19.7, 28.9)	<0.001	25.8 (51.7, 30.8)	<0.001	23.8 (20.0, 28.2)	<0.001	23.8 (20.1, 28.2)	<0.001
Poland	115	0.5	0.1	0.1	2.6%	71.7 (62.6, 82.1)	<0.001	76.4 (67.4, 85.6)	<0.001	73.4 (65.0, 83.0)	<0.001	73.5 (65.2, 82.9)	<0.001
Portugal	116	0.5	0.1	0.1	0.9%	41.9 (36.6, 48.0)	0.057	48.2 (42.5, 54.6)	0.54	46.6 (41.3, 52.7)	0.89	46.5 (41.3, 52.5)	0.90
Romania	119	0.5	0.1	0.1	5.1%	76.1 (66.4, 87.1)	<0.001	74.0 (65.4, 83.8)	<0.001	67.1 (59.4, 75.8)	<0.001	67.2 (59.5, 75.8)	<0.001
Sweden	97	0.3	0.3	0.3	1.0%	43.5 (38.0, 49.8)	0.18	49.9 (43.9, 56.6)	0.96	49.1 (43.5, 55.5)	0.32	49.0 (43.5, 55.3)	0.32
Slovenia	120	0.5	0.5	0.5	4.2%	48.8 (42.6, 55.9)	0.72	46.3 (40.8, 52.4)	0.21	40.5 (35.8, 45.7)	0.03	40.7 (36.1, 46.0)	0.03
Slovak Republic	127	2	0.61	0.24	22.0%	87.5 (76.4, 100.3)	<0.001	82.7 (73.0, 93.7)	<0.001	72.3 (64.0, 81.7)	<0.001	72.0 (63.8, 81.2)	<0.001
United Kingdom	21	0.5	0.5	0.5	14.3%	31.6 (27.6, 36.2)	<0.001	37.5 (33.0, 42.5)	<0.001	35.1 (31.0, 39.6)	<0.001	35.2 (31.2, 39.7)	<0.001

Urinary DEHP metabolites: sum of MEHP, 5OH-MEHP and 5oxo-MEHP; DEHP: di(2-ethylhexyl)phthalate; MEHP: mono(2-ethylhexyl)phthalate; LOQ: limit of quantification; GM: geometric mean; CI: confidence interval.

[a]Adjusted for age, gender and creatinine. [b]Adjusted for age and gender.

Table S27. Comparison of mean urinary level of DEHP metabolites between 17 European countries, results in mothers, unadjusted and adjusted data.

Country	N	LOQ MEHP (µg/L)	LOQ 5OH-MEHP (µg/L)	LOQ 5oxo-MEHP (µg/L)	% <LOQ	Urinary DEHP (µg/L) GM (95% CI), unadjusted	*p*-value	Urinary DEHP (µg/L) GM (95% CI), adjusted[a]	*p*-value	Urinary DEHP (µg/g cretainine) GM (95% CI), unadjusted	*p*-value	Urinary DEHP (µg/g creatinine) GM (95% CI), adjusted[b]	*p*-value
ALL	1800	0.3-3.9	0.1-9.2	0.1-6.2	18.4%	29.2 (28.1, 30.3)	<0.001	29.1 (28.1, 30.1)	<0.001	26.0 (25.2, 26.9)	<0.001	25.9 (25.0, 6.7)	<0.001
Belgium	125	0.5	0.1	0.1	7.2%	21.3 (18.3, 24.8)	<0.001	21.7 (19.0, 24.8)	<0.001	19.0 (16.7, 21.6)	<0.001	18.8 (16.5, 21.4)	<0.001
Switzerland	117	3.9	9.2	6.2	82.1%	18.4 (15.8, 21.4)	<0.001	20.4 (17.8, 23.3)	<0.001	19.0 (16.7, 21.6)	<0.001	18.7 (16.4, 21.3)	<0.001
Cyprus	59	2.6	2.5	1.6	42.4%	17.7 (14.3, 22.0)	<0.001	16.8 (13.9, 20.3)	<0.001	15.3 (12.7, 18.3)	<0.001	15.3 (12.7, 18.3)	<0.001
Czech Republic	117	2	0.61	0.24	23.9%	35.9 (30.8, 41.8)	0.006	37.3 (32.6, 42.6)	<0.001	33.8 (29.7, 38.4)	<0.001	33.9 (29.8, 38.6)	<0.001
Germany	116	0.5	0.2	0.2	15.5%	21.5 (18.5, 25.1)	<0.001	21.1 (18.5, 24.1)	<0.001	19.1 (16.8, 21.8)	<0.001	18.9 (16.6, 21.5)	<0.001
Denmark	143	0.14	0.91	0.67	8.4%	21.7 (18.6, 25.3)	<0.001	24.0 (21.0, 27.4)	0.003	22.2 (19.5, 25.2)	0.01	21.7 (19.0, 24.7)	0.006
Spain	118	0.5	0.2	0.2	0.0%	43.7 (37.5, 50.9)	<0.001	43.4 (38.0, 49.6)	<0.001	40.9 (35.9, 46.6)	<0.001	40.2 (35.2, 45.8)	<0.001
Hungary	115	2	0.61	0.24	25.2%	36.8 (31.5, 42.8)	0.002	34.0 (29.7, 38.8)	0.019	29.5 (25.9, 33.6)	0.049	29.8 (26.1, 34.0)	0.03
Ireland	120	1	0.5	0.5	10.0%	34.2 (29.3, 39.8)	0.036	32.3 (28.2, 36.8)	0.12	29.2 (25.7, 33.2)	0.07	29.1 (25.5, 33.1)	0.07
Luxembourg	56	0.5	0.1	0.1	13.3%	14.0 (11.3, 17.4)	<0.001	15.9 (13.2, 19.3)	<0.001	15.0 (12.5, 18.0)	<0.001	14.8 (12.3, 17.8)	<0.001
Poland	119	0.5	0.1	0.1	0.8%	43.1 (37.0, 50.3)	<0.001	43.9 (38.4, 50.2)	<0.001	39.3 (34.5, 44.7)	<0.001	39.7 (34.9, 45.2)	<0.001
Portugal	117	0.5	0.1	0.1	0.0%	39.9 (34.2, 46.5)	<0.001	37.2 (32.6, 42.5)	<0.001	32.0 (28.1, 36.5)	<0.001	32.1 (28.2, 36.5)	<0.001
Romania	117	0.5	0.1	0.1	5.1%	56.1 (48.2, 65.4)	<0.001	51.5 (45.0, 58.9)	<0.001	43.6 (38.3, 49.7)	<0.001	44.4 (39.0, 50.6)	<0.001
Sweden	96	0.3	0.3	0.3	5.2%	26.3 (22.6, 30.7)	0.17	28.4 (24.8, 32.4)	0.78	24.9 (21.9, 28.3)	0.50	24.7 (21.7, 28.2)	0.49
Slovenia	119	0.5	0.5	0.5	3.4%	30.9 (26.6, 36.1)	0.44	28.1 (24.6, 32.1)	0.58	24.0 (21.1, 27.3)	0.22	23.8 (20.9, 27.1)	0.19
Slovak Republic	125	2	0.61	0.24	20.0%	41.4 (35.5, 48.2)	<0.001	39.4 (34.5, 45.0)	<0.001	33.6 (29.5, 38.3)	<0.001	33.8 (29.7, 37.5)	<0.001
United Kingdom	21	0.5	0.5	0.5	23.8%	15.0 (12.9, 17.5)	<0.001	15.5 (13.5, 17.8)	<0.001	14.4 (12.6, 16.3)	<0.001	14.0 (12.3, 16.0)	<0.001

Urinary DEHP metabolites: sum of MEHP, 5OH-MEHP and 5oxo-MEHP; DEHP: di(2-ethylhexyl)phthalate; MEHP: mono(2-ethylhexyl)phthalate; LOQ: limit of quantification; GM: geometric mean; CI: confidence interval.

[a]Adjusted for age and creatinine. [b]Adjusted for age.

Table S28. Comparison of mean urinary MEP levels between 17 European countries, results in children, unadjusted and adjusted data.

Country	N	LOQ (µg/L)	% <LOQ	Urinary MEP (µg/L) GM (95% CI), unadjusted	*p*-value	Urinary MEP (µg/L)GM (95% CI), adjusted[a]	*p*-value	Urinary MEP (µg/g creatinine) GM (95% CI), unadjusted	*p*-value	Urinary MEP (µg/g creatinine) GM (95% CI), adjusted[b]	*p*-value
ALL	1816	0.5-11	2.0%	34.36 (32.78, 36.02)	<0.001	36.17 (34.50, 37.93)	<0.001	33.37 (31.93, 34.88)	<0.001	33.29 (31.86, 34.79)	<0.001
Belgium	125	0.5	0.0%	26.18 (21.69, 31.62)	0.004	26.70 (22.28, 31.99)	<0.001	23.39 (19.60, 27.91)	<0.001	23.41 (19.63, 27.90)	<0.001
Switzerland	119	11	30.3%	18.78 (15.55, 22.68)	<0.001	19.70 (16.45, 23.59)	<0.001	18.06 (15.14, 21.55)	<0.001	17.93 (15.04, 21.37)	<0.001
Cyprus	60	4	0.0%	38.97 (29.85, 50.88)	0.35	41.16 (31.90, 53.11)	0.31	37.25 (29.01, 47.82)	0.38	37.25 (29.05, 47.76)	0.37
Czech Republic	120	0.64	0.0%	31.60 (26.17, 38.15)	0.37	34.36 (28.68, 41.15)	0.56	32.20 (26.99, 38.43)	0.68	32.14 (26.96, 38.32)	0.68
Germany	120	0.5	0.0%	22.69 (18.79, 27.40)	<0.001	23.08 (19.27, 27.65)	<0.001	20.62 (17.28, 24.60)	<0.001	20.45 (17.15, 24.39)	<0.001
Denmark	142	0.53	0.0%	20.13 (16.67, 24.31)	<0.001	22.13 (18.47, 26.51)	<0.001	20.93 (17.54, 24.97)	<0.001	21.00 (17.61, 25.04)	<0.001
Spain	119	0.5	0.0%	181.9 (150.6, 219.6)	<0.001	208.3 (173.7, 249.8)	<0.001	198.9 (166.7, 237.4)	<0.001	198.9 (166.8, 237.1)	<0.001
Hungary	117	0.64	0.0%	46.98 (38.91, 56.73)	<0.001	45.38 (37.90, 54.34)	0.01	41.56 (34.83, 49.59)	0.019	41.55 (34.85, 49.54)	0.011
Ireland	120	0.5	0.0%	41.75 (34.57, 50.41)	0.037	42.47 (35.46, 50.85)	0.07	38.66 (32.40, 46.13)	0.09	38.72 (32.47, 46.16)	0.08
Luxembourg	59	0.5	0.0%	24.68 (18.91, 32.22)	0.013	26.84 (20.81, 34.61)	0.019	24.60 (19.16, 31.58)	0.015	24.61 (19.19, 31.55)	0.015
Poland	115	0.5	0.0%	43.79 (36.27, 52.88)	0.009	46.89 (39.15, 56.16)	0.003	44.86 (37.60, 53.52)	<0.001	44.90 (37.66, 53.54)	<0.001
Portugal	116	0.5	0.0%	43.27 (35.84, 52.25)	0.013	50.17 (41.82, 60.18)	<0.001	48.16 (40.36, 57.46)	<0.001	48.06 (40.31, 57.29)	<0.001
Romania	119	0.5	0.0%	35.90 (29.73, 43.35)	0.64	34.83 (29.10, 41.69)	0.67	31.67 (26.54, 37.79)	0.55	31.65 (26.55, 37.73)	0.56
Sweden	97	0.3	0.0%	28.83 (23.87, 34.81)	0.06	33.34 (27.77, 40.03)	0.36	32.56 (27.29, 38.85)	0.78	32.49 (27.25, 38.73)	0.78
Slovenia	120	0.5	0.0%	43.22 (35.80, 52.19)	0.014	40.17 (33.53, 48.13)	0.24	35.88 (30.07, 42.81)	0.41	35.46 (29.72, 42.30)	0.47
Slovak Republic	127	0.64	0.0%	39.64 (32.83, 47.86)	0.12	37.53 (31.34, 44.93)	0.68	32.72 (27.42, 39.04)	0.82	32.70 (27.42, 38.98)	0.08
United Kingdom	21	0.5	0.0%	14.33 (11.87, 17.30)	<0.001	16.95 (14.12, 20.34)	<0.001	15.92 (13.34, 18.99)	<0.001	15.78 (13.23, 18.82)	<0.001

MEP: mono-ethyl phthalate; LOQ: limit of quantification; GM: geometric mean; CI: confidence interval.

[a]Adjusted for age, gender and creatinine. [b]Adjusted for age and gender

Table S29. Comparison of mean urinary MEP levels between 17 European countries, results in mothers, unadjusted and adjusted data.

Country	N	LOQ (µg/L)	% <LOQ	Urinary MEP (µg/L) GM (95% CI), unadjusted	*p*-value	Urinary MEP (µg/L) GM (95% CI), adjusted[a]	*p*-value	Urinary MEP (µg/g creatinine) GM (95% CI), unadjusted	*p*-value	Urinary MEP (µg/g creatinine) GM (95% CI), adjusted[b]	*p*-value
ALL	1800	0.5-11	4.8%	48.20 (45.59, 50.97)	<0.001	48.09 (45.59, 50.73)	<0.001	42.94 (40.75, 45.24)	<0.001	42.77 (40.56, 45.11)	<0.001
Belgium	125	0.5	0.0%	36.30 (29.04, 45.38)	0.010	37.10 (30.06, 45.77)	0.012	32.39 (26.27, 39.92)	0.006	32.16 (26.06, 39.68)	0.012
Switzerland	117	11	25.6%	28.13 (22.51, 35.17)	<0.001	31.18 (25.24, 38.52)	<0.001	29.10 (23.60, 35.87)	<0.001	28.52 (23.10, 35.21)	0.006
Cyprus	59	4	1.7%	92.42 (67.41, 126.7)	<0.001	87.68 (65.21, 117.9)	<0.001	79.59 (59.21, 107.0)	<0.001	79.68 (59.28, 107.1)	<0.001
Czech Republic	117	0.64	0.0%	56.66 (45.32, 70.82)	0.15	59.17 (47.97, 73.00)	0.046	53.28 (43.23, 65.68)	0.037	53.77 (43.59, 66.33)	0.027
Germany	116	0.5	0.0%	39.41 (31.53, 49.27)	0.07	38.55 (31.24, 47.57)	0.033	34.97 (28.37, 43.11)	0.047	34.46 (27.92, 42.51)	0.037
Denmark	143	0.53	0.0%	33.74 (26.99, 42.18)	<0.001	37.29 (30.17, 46.08)	0.015	34.43 (27.94, 42.45)	0.033	33.70 (27.28, 41.64)	0.022
Spain	118	0.5	0.0%	161.1 (128.9, 201.4)	<0.001	160.0 (129.5, 197.6)	<0.001	150.8 (122.4, 185.9)	<0.001	148.0 (119.8, 182.8)	<0.001
Hungary	115	0.64	0.0%	55.04 (44.03, 68.80)	0.23	50.88 (41.24, 62.78)	0.59	44.15 (35.82, 54.42)	0.79	44.70 (36.24, 55.13)	0.67
Ireland	120	0.5	0.0%	58.72 (46.98, 73.40)	0.07	55.25 (44.80, 68.13)	0.18	50.15 (40.69, 61.82)	0.13	49.91 (40.49, 61.53)	0.14
Luxembourg	56	0.5	0.0%	31.77 (23.17, 43.56)	0.009	36.38 (26.96, 49.08)	0.06	34.11 (25.37, 45.85)	0.12	33.81 (25.10, 45.53)	0.11
Poland	119	0.5	0.0%	41.66 (33.33, 52.08)	0.19	42.52 (34.47, 52.45)	0.24	37.94 (30.78, 46.76)	0.23	38.43 (31.16, 47.40)	0.30
Portugal	117	0.5	0.0%	59.84 (47.87, 74.80)	0.049	55.85 (45.28, 68.90)	0.15	48.05 (38.98, 59.22)	0.28	48.31 (39.18, 59.58)	0.24
Romania	117	0.5	0.0%	48.03 (38.42, 60.04)	0.97	44.23 (35.79, 54.66)	0.43	37.34 (30.29, 46.03)	0.18	38.23 (30.95, 47.23)	0.28
Sweden	96	0.3	0.0%	43.05 (34.44, 53.81)	0.30	46.53 (37.71, 57.42)	0.75	40.69 (33.01, 50.16)	0.60	40.57 (32.90, 50.04)	0.61
Slovenia	119	0.5	0.0%	51.75 (41.40, 64.69)	0.52	46.82 (37.94, 57.77)	0.80	40.17 (32.59, 49.51)	0.52	39.85 (32.31, 49.14)	0.49
Slovak Republic	125	0.64	0.0%	54.81 (43.84, 68.51)	0.24	52.21 (42.33, 64.39)	0.43	44.53 (36.12, 54.89)	0.72	44.91 (36.42, 55.37)	0.64
United Kingdom	21	0.5	0.0%	26.75 (21.40, 33.44)	<0.001	27.39 (22.11, 33.92)	<0.001	25.63 (20.80, 31.60)	<0.001	24.79 (20.02, 30.70)	<0.001

MEP: mono-ethyl phthalate; LOQ: limit of quantification; GM: geometric mean; CI:confidence interval.

[a]Adjusted for age and creatinine. [b]Adjusted for age.

Table S30. Comparison of mean urinary MBzP levels between 17 European countries, results in children, unadjusted and adjusted data.

Country	N	LOQ (μg/L)	% <LOQ	Urinary MBzP (μg/L) GM (95% CI), unadjusted	*p*-value	Urinary MBzP (μg/L) GM (95% CI), adjusted[a]	*p*-value	Urinary MBzP (μg/g creatinine) GM (95% CI), unadjusted	*p*-value	Urinary MBzP (μg/g creatinine) GM (95% CI), adjusted[b]	*p*-value
ALL	1816	0.2-1.9	4.8%	7.15 (6.83, 7.48)	<0.001	7.56 (7.22, 7.91)	<0.001	6.94 (6.66, 7.24)	<0.001	6.94 (6.66, 7.24)	<0.001
Belgium	125	0.2	0.0%	8.78 (7.33, 10.5)	0.021	8.96 (7.54, 10.6)	0.044	7.84 (6.62, 9.29)	0.15	7.83 (6.62, 9.28)	0.15
Switzerland	119	5	57.1%	4.84 (4.04, 5.79)	<0.001	5.10 (4.30, 6.06)	<0.001	4.65 (3.92, 5.51)	<0.001	4.64 (3.92, 5.49)	<0.001
Cyprus	60	1.9	16.7%	3.48 (2.70, 4.49)	<0.001	3.69 (2.89, 4.70)	<0.001	3.33 (2.62, 4.23)	<0.001	3.33 (2.62, 4.23)	<0.001
Czech Republic	120	1.2	0.0%	8.32 (6.94, 9.96)	0.09	9.06 (7.63, 10.8)	0.032	8.48 (7.15, 10.0)	0.017	8.47 (7.15, 10.0)	0.018
Germany	120	0.2	0.0%	6.47 (5.40, 7.75)	0.26	6.59 (5.54, 7.82)	0.10	5.88 (4.97, 6.97)	0.048	5.82 (4.92, 6.89)	0.034
Denmark	142	1.14	2.8%	7.21 (6.02, 8.64)	0.92	7.95 (6.69, 9.45)	0.55	7.50 (6.33, 8.89)	0.36	7.54 (6.37, 8.93)	0.33
Spain	119	0.2	0.0%	12.7 (10.6, 15.2)	<0.001	14.6 (12.2, 17.3)	<0.001	13.9 (11.7, 16.5)	<0.001	13.9 (11.7, 16.4)	<0.001
Hungary	117	1.2	0.9%	7.57 (6.32, 9.07)	0.52	7.32 (6.17, 8.70)	0.71	6.70 (5.65, 7.94)	0.67	6.69 (5.65, 7.92)	0.66
Ireland	120	0.5	0.0%	5.83 (4.87, 6.98)	0.022	5.92 (4.99, 7.03)	0.004	5.40 (4.56, 6.40)	0.003	5.39 (4.55, 6.38)	0.002
Luxembourg	59	0.2	0.0%	4.75 (3.68, 6.14)	<0.001	5.18 (4.06, 6.60)	0.002	4.74 (3.73, 6.02)	<0.001	4.75 (3.74, 6.03)	<0.001
Poland	115	0.5	0.9%	8.71 (7.27, 10.4)	0.027	9.33 (7.86, 11.1)	0.013	8.92 (7.53, 10.6)	0.003	8.93 (7.54, 10.6)	0.003
Portugal	116	0.2	0.0%	6.98 (5.83, 8.36)	0.79	8.11 (6.82, 9.65)	0.41	7.77 (6.56, 9.20)	0.18	7.76 (6.55, 9.18)	0.18
Romania	119	0.2	1.7%	4.26 (3.55, 5.10)	<0.001	4.14 (3.49, 4.91)	<0.001	3.76 (3.17, 4.45)	<0.001	3.76 (3.18, 4.45)	<0.001
Sweden	97	0.3	0.0%	19.9 (16.6, 23.8)	<0.001	23.1 (19.4, 27.5)	<0.001	22.5 (19.0, 26.6)	<0.001	22.5 (19.0, 26.6)	<0.001
Slovenia	120	0.5	0.0%	8.34 (6.96, 9.98)	0.09	7.95 (6.69, 9.45)	0.55	6.92 (5.84, 8.20)	0.97	7.01 (5.92, 8.31)	0.91
Slovak Republic	127	1.2	0.8%	8.35 (6.97, 10.0)	0.08	7.89 (6.65, 9.37)	0.61	6.89 (5.82, 8.17)	0.93	6.86 (5.80, 8.13)	0.89
United Kingdom	21	0.5	0.0%	3.46 (2.89, 4.14)	<0.001	4.15 (3.49, 4.94)	<0.001	3.84 (3.24, 4.55)	<0.001	3.87 (3.26, 4.58)	<0.001

MBzP: mono-benzyl phthalate; LOQ: limit of quantification; GM: geometric mean; CI:confidence interval.

[a]Adjusted for age, gender and creatinine. [b]Adjusted for age and gender.

Table S31. Comparison of mean urinary MBzP levels between 17 European countries, results in mothers, unadjusted and adjusted data.

Country	N	LOQ (µg/L)	% <LOQ	Urinary MBzP (µg/L) GM (95% CI), unadjusted	*p*-value	Urinary MBzP (µg/L) GM (95% CI), adjusted[a]	*p*-value	Urinary MBzP (µg/g creatinine) GM (95% CI), unadjusted	*p*-value	Urinary MBzP (µg/g creatinine) GM (95% CI), adjusted[b]	*p*-value
ALL	1800	0.2-5	8.2%	4.51 (4.31, 4.72)	<0.001	4.48 (4.30, 4.67)	<0.001	4.02 (3.87, 4.18)	<0.001	3.98 (3.83, 4.14)	<0.001
Belgium	125	0.2	0.0%	6.47 (5.41, 7.74)	<0.001	6.54 (5.58, 7.68)	<0.001	5.77 (4.94, 6.74)	<0.001	5.67 (4.85, 6.62)	<0.001
Switzerland	117	5	72.6%	3.47 (2.90, 4.15)	0.003	3.90 (3.32, 4.58)	0.08	3.59 (3.07, 4.19)	0.14	3.55 (3.04, 4.15)	0.13
Cyprus	59	1.9	32.2%	2.54 (1.97, 3.27)	<0.001	2.39 (1.91, 2.99)	<0.001	2.19 (1.76, 2.73)	<0.001	2.18 (1.75, 2.71)	<0.001
Czech Republic	117	1.2	9.4%	4.62 (3.87, 5.53)	0.78	4.75 (4.05, 5.57)	0.46	4.35 (3.72, 5.08)	0.30	4.30 (3.68, 5.03)	0.31
Germany	116	0.2	0.0%	4.55 (3.80, 5.44)	0.93	4.46 (3.80, 5.23)	0.95	4.03 (3.45, 4.71)	0.96	3.99 (3.41, 4.66)	0.99
Denmark	143	1.14	7.0%	4.00 (3.35, 4.79)	0.18	4.46 (3.80, 5.24)	0.96	4.09 (3.50, 4.77)	0.82	4.01 (3.43, 4.69)	0.92
Spain	118	0.2	0.0%	8.53 (7.13, 10.2)	<0.001	8.45 (7.20, 9.93)	<0.001	7.99 (6.84, 9.33)	<0.001	7.83 (6.70, 9.16)	<0.001
Hungary	115	1.2	5.2%	4.76 (3.98, 5.70)	0.54	4.34 (3.70, 5.09)	0.69	3.82 (3.27, 4.46)	0.51	3.82 (3.27, 4.47)	0.59
Ireland	120	0.5	2.5%	3.65 (3.05, 4.36)	0.017	3.43 (2.92, 4.02)	<0.001	3.12 (2.67, 3.64)	<0.001	3.10 (2.66, 3.62)	<0.001
Luxembourg	56	0.2	0.0%	3.15 (2.44, 4.06)	0.005	3.55 (2.83, 4.46)	0.042	3.38 (2.71, 4.21)	0.12	3.27 (2.62, 4.08)	0.07
Poland	119	0.5	0.8%	4.49 (3.75, 5.37)	0.95	4.55 (3.88, 5.33)	0.85	4.09 (3.50, 4.77)	0.83	4.10 (3.51, 4.79)	0.70
Portugal	117	0.2	0.0%	6.11 (5.11, 7.31)	<0.001	5.61 (4.78, 6.58)	0.004	4.91 (4.20, 5.73)	0.009	4.86 (4.17, 5.68)	0.009
Romania	117	0.2	2.6%	2.78 (2.32, 3.32)	<0.001	2.49 (2.12, 2.93)	<0.001	2.16 (1.85, 2.52)	<0.001	2.16 (1.85, 2.53)	<0.001
Sweden	96	0.3	0.0%	12.9 (10.7, 15.4)	<0.001	13.8 (11.7, 16.2)	<0.001	12.1 (10.4, 14.2)	<0.001	12.0 (10.2, 14.0)	<0.001
Slovenia	119	0.5	0.0%	4.95 (4.13, 5.92)	0.30	4.44 (3.78, 5.21)	0.90	3.84 (3.29, 4.48)	0.55	3.79 (3.25, 4.43)	0.52
Slovak Republic	125	1.2	4.8%	4.70 (3.93, 5.62)	0.65	4.41 (3.76, 5.18)	0.85	3.81 (3.27, 4.45)	0.50	3.80 (3.26, 4.44)	0.54
United Kingdom	21	0.5	14.3%	1.63 (1.36, 1.95)	<0.001	1.74 (1.48, 2.05)	<0.001	1.56 (1.34, 1.82)	<0.001	1.57 (1.34, 1.84)	<0.001

MBzP: mono-benzyl phthalate; LOQ: limit of quantification; GM: geometric mean; CI: confidence interval.

[a]Adjusted for age and creatinine. [b]Adjusted for age.

Table S32. Comparison of mean urinary MnBP levels between 17 European countries, results in children, unadjusted and adjusted data.

Country	N	LOQ (µg/L)	% <LOQ	Urinary MnBP (µg/L) GM (95% CI), unadjusted	*p*-value	Urinary MnBP (µg/L) GM (95% CI), adjusted[a]	*p*-value	Urinary MnBP (µg/g creatinine) GM (95% CI), unadjusted	*p*-value	Urinary MnBP (µg/g creatinine) GM (95% CI), adjusted[b]	*p*-value
ALL	1355	0.5-4.4	0.1%	34.8 (33.5, 36.2)	<0.001	36.6 (35.3, 38.0)	<0.001	34.0 (32.8, 35.2)	<0.001	33.9 (32.8, 35.1)	<0.001
Belgium	125	0.5	0.0%	39.0 (34.1, 44.6)	0.09	39.4 (34.9, 44.6)	0.22	34.8 (30.8, 39.3)	0.68	34.8 (30.9, 39.3)	0.65
Switzerland	119	4.4	1.7%	19.4 (16.9, 22.1)	<0.001	20.1 (17.8, 22.8)	<0.001	18.6 (16.5, 21.0)	<0.001	18.4 (16.4, 20.8)	<0.001
Cyprus	60	1.6	0.0%	19.7 (16.3, 23.8)	<0.001	20.6 (17.3, 24.6)	<0.001	18.8 (15.9, 22.4)	<0.001	18.8 (15.9, 22.3)	<0.001
Germany	120	1	0.0%	46.1 (40.3, 52.7)	<0.001	46.4 (41.0, 52.5)	<0.001	41.9 (37.1, 47.3)	<0.001	41.4 (36.7, 46.6)	<0.001
Denmark	142	1.43	0.0%	30.8 (26.9, 35.3)	0.06	33.6 (29.7, 38.1)	0.15	32.0 (28.4, 36.2)	0.32	32.2 (28.6, 36.3)	0.37
Spain	119	1	0.0%	46.6 (40.7, 53.3)	<0.001	52.7 (46.5, 59.7)	<0.001	51.0 (45.1, 57.5)	<0.001	50.9 (45.2, 57.4)	<0.001
Ireland	120	1	0.0%	28.2 (24.6, 32.2)	<0.001	28.5 (25.2, 32.2)	<0.001	26.1 (23.1, 29.5)	<0.001	26.1 (23.2, 29.4)	<0.001
Luxembourg	59	0.5	0.0%	26.1 (21.6, 31.6)	0.002	28.2 (23.7, 33.6)	0.003	26.0 (21.9, 30.9)	0.002	26.1 (22.0, 30.9)	0.002
Poland	115	0.5	0.0%	85.1 (74.4, 97.4)	<0.001	90.4 (80.0, 102)	<0.001	87.2 (77.2, 98.4)	<0.001	87.3 (77.5, 98.4)	<0.001
Portugal	116	0.5	0.0%	29.1 (25.5, 33.3)	0.007	33.3 (29.4, 37.7)	0.11	32.4 (28.7, 36.6)	0.43	32.3 (28.7, 36.4)	0.41
Romania	119	0.5	0.0%	44.4 (38.8, 50.8)	<0.001	43.2 (38.2, 48.8)	0.007	39.2 (34.7, 44.2)	0.015	39.2 (34.8, 44.1)	0.014
Slovenia	120	0.5	0.0%	40.0 (34.9, 45.7)	0.036	38.0 (33.5, 42.9)	0.56	33.2 (29.4, 37.5)	0.69	33.4 (29.6, 37.7)	0.79
United Kingdom	21	0.5	0.0%	22.6 (19.8, 25.9)	<0.001	26.4 (23.3, 29.9)	<0.001	25.1 (22.3, 28.4)	<0.001	24.9 (22.1, 28.1)	<0.001

MnBP: mono-n-butyl phthalate; LOQ: limit of quantification; GM: geometric mean; CI:confidence interval.

[a]Adjusted for age, gender and creatinine. [b]Adjusted for age and gender.

Table S33. Comparison of mean urinary MnBP levels between 17 European countries, results in mothers, unadjusted and adjusted data.

Country	N	LOQ (µg/L)	% <LOQ	Urinary MnBP (µg/L) GM (95% CI), unadjusted	*p*-value	Urinary MnBP (µg/L) GM (95% CI), adjusted[a]	*p*-value	Urinary MnBP (µg/g creatinine) GM (95% CI), unadjusted	*p*-value	Urinary MnBP (µg/g creatinine) GM (95% CI), adjusted[b]	*p*-value
ALL	1347	0.5-4.4	0.6%	23.9 (23.0, 24.9)	<0.001	23.4 (22.6, 24.2)	<0.001	21.5 (20.8, 22.2)	<0.001	20.8 (20.1, 21.6)	<0.001
Belgium	125	0.5	0.0%	30.9 (26.8, 35.6)	<0.001	30.5 (27.1, 34.4)	<0.001	27.5 (24.4, 31.0)	<0.001	26.5 (23.5, 29.8)	<0.001
Switzerland	117	4.4	6.8%	13.2 (11.4, 15.2)	<0.001	13.9 (12.3, 15.7)	<0.001	13.6 (12.1, 15.3)	<0.001	12.7 (11.3, 14.3)	<0.001
Cyprus	59	1.6	0.0%	17.0 (13.9, 20.8)	<0.001	16.1 (13.6, 19.1)	<0.001	14.7 (12.4, 17.5)	<0.001	14.6 (12.4, 17.3)	<0.001
Germany	116	1	0.0%	31.5 (27.3, 36.3)	<0.001	29.7 (26.4, 33.5)	<0.001	27.9 (24.8, 31.5)	<0.001	26.5 (23.6, 29.8)	<0.001
Denmark	143	1.43	0.0%	20.7 (18.0, 23.9)	0.038	21.6 (19.2, 24.4)	0.19	21.2 (18.8, 23.9)	0.80	19.6 (17.4, 22.1)	0.28
Spain	118	1	0.0%	32.7 (28.4, 37.7)	<0.001	30.8 (27.3, 34.7)	<0.001	30.6 (27.1, 34.5)	<0.001	28.5 (25.3, 32.0)	<0.001
Ireland	120	1	0.0%	21.7 (18.8, 25.0)	0.15	20.2 (18.0, 22.8)	0.014	18.5 (16.4, 20.9)	0.011	18.2 (16.2, 20.5)	0.019
Luxembourg	56	0.5	0.0%	16.8 (13.7, 20.5)	<0.001	18.3 (15.4, 21.7)	0.004	18.0 (15.2, 21.3)	0.036	16.9 (14.3, 20.0)	0.013
Poland	119	0.5	0.0%	46.0 (40.0, 53.0)	<0.001	48.2 (42.8, 54.4)	<0.001	41.9 (37.2, 47.2)	<0.001	43.6 (38.7, 49.0)	<0.001
Portugal	117	0.5	0.0%	23.8 (20.7, 27.4)	0.93	22.3 (19.8, 25.1)	0.42	19.1 (17.0, 21.5)	0.045	19.2 (17.1, 21.6)	0.15
Romania	117	0.5	0.0%	28.2 (24.5, 32.5)	0.018	27.1 (24.0, 30.6)	0.014	21.9 (19.5, 24.7)	0.72	23.4 (20.8, 26.3)	0.05
Slovenia	119	0.5	0.0%	26.9 (23.3, 31.0)	0.09	23.8 (21.1, 26.8)	0.76	20.9 (18.5, 23.5)	0.61	20.1 (17.9, 22.7)	0.55
United Kingdom	21	0.5	0.0%	13.5 (11.7, 15.5)	<0.001	13.1 (11.6, 14.9)	<0.001	12.9 (11.4, 14.5)	<0.001	11.9 (10.5, 13.4)	<0.001

MnBP: mono-n-butyl phthalate; LOQ: limit of quantification; GM: geometric mean; CI: confidence interval.

[a]Adjusted for age and creatinine. [b]Adjusted for age.

Table S34. Comparison of mean urinary MiBP levels between 17 European countries, results in children, unadjusted and adjusted data.

**Country**	**N**	**LOQ (µg/L)**	**% <LOQ**	**Urinary MiBP (µg/L) GM (95% CI), unadjusted**	***p*-value**	**Urinary MiBP (µg/L) GM (95% CI), adjusted[a]**	***p*-value**	**Urinary MiBP (µg/g creatinine) GM (95% CI), unadjusted**	***p*-value**	**Urinary MiBP (µg/g creatinine) GM (95% CI), adjusted[b]**	***p*-value**
ALL	1355	0.5-4.9	0.2%	45.42 (43.59, 47.32)	<0.001	47.94 (46.07, 49.89)	<0.001	44.31 (42.69, 46.00)	<0.001	44.21 (42.62, 45.87)	<0.001
Belgium	125	0.5	0.0%	58.16 (50.45, 67.05)	<0.001	59.20 (51.88, 67.54)	<0.001	51.96 (45.65, 59.13)	0.01	51.98 (45.76, 59.05)	0.009
Switzerland	119	4.9	2.5%	19.67 (17.07, 22.68)	<0.001	20.54 (18.01, 23.41)	<0.001	18.92 (16.63, 21.53)	<0.001	18.74 (16.50, 21.29)	<0.001
Cyprus	60	1.4	0.0%	49.16 (40.21, 60.11)	0.43	51.75 (42.98, 62.32)	0.41	46.99 (39.13, 56.42)	0.52	46.99 (39.24, 56.26)	0.50
Germany	120	1	0.0%	40.95 (35.52, 47.21)	0.14	41.40 (36.29, 47.23)	0.02	37.22 (32.70, 42.36)	0.006	36.76 (32.36, 41.75)	0.003
Denmark	142	1.1	0.0%	56.76 (49.24, 65.43)	<0.001	62.22 (54.54, 70.99)	<0.001	59.01 (51.85, 67.16)	<0.001	59.32 (52.23, 67.38)	<0.001
Spain	119	1	0.0%	56.12 (48.68, 64.70)	0.002	63.83 (55.91, 72.88)	<0.001	61.38 (53.93, 69.86)	<0.001	61.35 (54.01, 69.69)	<0.001
Ireland	120	1	0.0%	44.67 (38.75, 51.49)	0.81	45.36 (39.78, 51.73)	0.39	41.37 (36.35, 47.08)	0.28	41.41 (36.46, 47.04)	0.29
Luxembourg	59	0.5	0.0%	34.03 (27.83, 41.61)	0.004	36.88 (30.64, 44.39)	0.005	33.92 (28.25, 40.73)	0.003	33.95 (28.35, 40.65)	0.003
Poland	115	0.5	0.0%	101.5 (88.03, 117.0)	<0.001	108.3 (94.96, 123.5)	<0.001	103.9 (91.34, 118.3)	<0.001	104.1 (91.64, 118.2)	<0.001
Portugal	116	0.5	0.0%	35.07 (30.42, 40.43)	<0.001	40.33 (35.31, 46.06)	0.007	39.03 (34.29, 44.42)	0.04	38.92 (34.26, 44.21)	0.04
Romania	119	0.5	0.0%	52.60 (45.63, 60.64)	0.03	51.11 (44.84, 58.26)	0.32	46.40 (40.77, 52.81)	0.46	46.38 (40.83, 52.68)	0.44
Slovenia	120	1	0.0%	58.37 (50.63, 67.29)	<0.001	54.98 (48.20, 62.70)	0.03	48.46 (42.58, 55.15)	0.16	48.38 (42.57, 54.99)	0.15
United Kingdom	21	1	0.0%	25.83 (22.41, 29.78)	<0.001	30.31 (26.53, 34.64)	<0.001	28.70 (25.21, 32.66)	<0.001	28.47 (25.06, 32.35)	<0.001

MiBP: mono-iso-butyl phthalate; LOQ: limit of quantification; GM: geometric mean; CI: confidence interval.

[a]Adjusted for age, gender and creatinine. [b]Adjusted for age and gender.

Table S35. Comparison of mean urinary MiBP levels between 17 European countries, results in mothers, unadjusted and adjusted data.

Country	N	LOQ (µg/L)	% <LOQ	Urinary MiBP (µg/L) GM (95% CI), unadjusted	*p*-value	Urinary MiBP (µg/L) GM (95% CI), adjusted[a]	*p*-value	Urinary MiBP (µg/g creatinine) GM (95% CI), unadjusted	*p*-value	Urinary MiBP (µg/g creatinine) GM (95% CI), adjusted[b]	*p*-value
ALL	1347	0.5-4.9	0.6%	30.12 (28.92, 31.37)	<0.001	30.07 (28.99, 31.18)	<0.001	27.02 (26.10, 27.96)	<0.001	26.74 (25.81, 27.71)	<0.001
Belgium	125	0.5	0.0%	38.08 (33.07, 43.84)	<0.001	38.60 (34.13, 43.65)	<0.001	33.97 (30.16, 38.28)	<0.001	33.49 (29.71, 37.76)	<0.001
Switzerland	117	4.9	6.8%	13.09 (11.37, 15.07)	<0.001	14.41 (12.73, 16.31)	<0.001	13.54 (12.02, 15.25)	<0.001	13.26 (11.75, 14.95)	<0.001
Cyprus	59	1.4	0.0%	46.01 (37.69, 56.16)	<0.001	43.70 (36.76, 51.95)	<0.001	39.62 (33.47, 46.90)	<0.001	39.56 (33.42, 46.81)	<0.001
Germany	116	1	0.0%	25.07 (21.78, 28.87)	0.008	24.61 (21.76, 27.83)	<0.001	22.25 (19.74, 25.06)	<0.001	21.87 (19.40, 24.66)	<0.001
Denmark	143	1.1	0.0%	37.99 (32.99, 43.74)	<0.001	41.56 (36.70, 47.06)	<0.001	38.77 (34.41, 43.68)	<0.001	37.79 (33.48, 42.64)	<0.001
Spain	118	1	0.0%	37.42 (32.50, 43.09)	0.002	36.97 (32.66, 41.84)	<0.001	35.03 (31.09, 39.47)	<0.001	34.19 (30.30, 38.58)	<0.001
Ireland	120	1	0.0%	27.85 (24.19, 32.07)	0.26	26.46 (23.41, 29.91)	0.03	23.79 (21.11, 26.80)	0.03	23.66 (21.00, 26.65)	0.04
Luxembourg	56	0.5	0.0%	18.53 (15.19, 22.62)	<0.001	21.07 (17.67, 25.12)	<0.001	19.90 (16.81, 23.55)	<0.001	19.45 (16.41, 23.06)	<0.001
Poland	119	0.5	0.0%	52.54 (45.63, 60.49)	<0.001	53.64 (47.44, 60.64)	<0.001	57.84 (42.46, 53.90)	<0.001	48.42 (42.96, 54.57)	<0.001
Portugal	117	0.5	0.0%	30.21 (26.24, 34.79)	0.96	28.40 (25.13, 32.11)	0.34	24.26 (21.53, 27.33)	0.06	24.26 (21.53, 27.34)	0.10
Romania	117	0.5	0.0%	37.57 (32.63, 43.26)	<0.001	34.66 (30.61, 39.23)	0.02	29.21 (25.93, 32.91)	0.18	29.77 (26.39, 33.59)	0.07
Slovenia	119	1	0.0%	38.11 (33.10, 43.88)	<0.001	34.87 (30.83, 39.43)	0.01	29.58 (26.25, 33.32)	0.12	29.22 (25.93, 32.93)	0.13
United Kingdom	21	1	0.0%	16.98 (14.75, 19.55)	<0.001	17.57 (15.59, 19.92)	<0.001	16.27 (14.44, 18.33)	<0.001	15.89 (14.06, 17.96)	<0.001

MiBP: mono-iso-butyl phthalate; LOQ: limit of quantification; GM: geometric mean; CI: confidence interval.

[a]Adjusted for age and creatinine. [b]Adjusted for age.
